# Evaluation of plant diversity and soil-vegetation relationships in some salinity-affected cultivated areas in Egypt

**DOI:** 10.1371/journal.pone.0346662

**Published:** 2026-04-24

**Authors:** Entesar Abou Glida, Abdel Aziz Mohamed Ragab, Om Mohamed Ahmed Khafagi, Ramadan Bedair

**Affiliations:** 1 Botany and Microbiology Department, Faculty of Science (Girls Branch), Al-Azhar University, Cairo, Egypt; 2 Soil, Water and Environment Research Institute, Agriculture Research Center, Giza, Egypt; 3 Botany and Microbiology Department, Faculty of Science, Al-Azhar University, Nasr City, Cairo, Egypt; University of Balochistan, PAKISTAN

## Abstract

Soil salinity, characterized by the accumulation of soluble salts, poses a significant global threat to agriculture, affecting over one billion hectares. It induces osmotic stress and ion toxicity, significantly diminishing crop yields and arable land. Egypt, heavily reliant on the Nile Delta, experiences severe salinization resulting from irrigation practices, climate change, and inherent aridity. This escalating crisis compromises agricultural productivity and food security, necessitating urgent global solutions. A total of 103 plant taxa were documented. Asteraceae (22 species) and Poaceae (13 species) constituted the most diverse families. Annuals (50.0%) and therophytes (49.5%) represented the predominant life forms, while biregional species (36.9%) formed the largest chorological group. Soil physicochemical properties, including particle size distribution, pH, EC, TDS, organic matter, saturation percentage, SAR, available N, P, and K, major ions (Ca² ⁺ , Mg² ⁺ , Na ⁺ , K ⁺ , Cl ⁻ , HCO₃ ⁻ , SO₄²⁻), and CaCO₃%, were determined in all stands. Two-Way Indicator Species Analysis (TWINSPAN) and Detrended Correspondence Analysis (DCA) classified the studied stands into six distinct groups. Stands within each group exhibited ecological similarity. Each vegetation group possessed its own set of indicator plant species, and the soil factors most closely associated with them.

## Introduction

Agroecosystems cover 40% of the Earth’s land surface. They provide environmental services, including food and non-food biomass production, carbon sequestration, nutrient cycling, and climate regulation. They are important habitats for many terrestrial plants and animal species [1]. The FAO defines soil health as “the capacity of soils to support the productivity, diversity, and environmental services of terrestrial ecosystems [2]. Plant diversity plays a pivotal role in sustaining ecosystem services such as carbon sequestration, nutrient cycling, and soil fertility [3]. Functional diversity, particularly traits associated with resource acquisition, enhances these services by optimizing biomass production and organic matter retention. Agroecological practices, including agroforestry and organic farming, bolster biodiversity, thereby improving ecosystem resilience and reducing dependency on synthetic inputs [4]. Ecological intensification through diversified cropping systems and integration of non-crop vegetation supports pollinator habitats and natural pest regulation, fostering productive and sustainable agroecosystems [5].

The management practices used in agroecosystems determine the state of the global environment [1]. Each year, approximately 1–2% of the world’s cultivated soils are degraded by salinity, and around 23% of arable land (800 million hectares) is affected by salinity, posing a significant threat to food production [6]. Soil salinity stands as one of the most serious threats to Earth’s agricultural production, especially in arid and semiarid regions, where salt-affected soils are commonly found [7]. On a global scale, it is estimated that 50% of arable land will become salt-affected by the end of 2050. This is due to rising groundwater levels with high salt concentrations, inefficient irrigation and drainage systems, and excessive use of chemical fertilizers [8].

These conditions are caused either by natural events or by irrigation with saline waters [9,10]. The increased concentration of soluble salts in soils is considered the primary cause of soil salinization, disrupting ecosystem functions and limiting crop growth and productivity [11]. Poor vegetation cover and excessive use of irrigation water and agricultural inputs also contribute to groundwater imbalances and soil salinization [12,13], reliance on wastewater for irrigation [14–16], and is a major threat to global coastal ecosystems [17]. Saltwater intrusion into coastal areas [13]. All of this leads to increased salinity in agricultural lands [14–16]. The negative impact of various types of salts on soil properties limits crop production [18–20]. Soil salinity significantly reduces crop productivity by hindering plant growth, nutrient uptake, and water balance, and is primarily caused by salt stress [21]. According to FAO estimates, the area of salt-affected soil is estimated at 1,381 million hectares, equivalent to 10.7% of the world’s total land area. The organization also estimates that 10% of irrigated cropland and 10% of rainfed cropland are affected by salinity [22]. 33% of the world’s irrigated land is affected by soil salinity, and more than 50% of arable land is expected to become saline by 2050. Global drought trend models indicate that the current trend of rising temperatures could increase the total land area affected by salt by 24–32%. Most droughts are expected to occur in developing countries [22]. Egyptian agroecosystems predominantly cultivate wheat, berseem, cotton, rice, and maize, with spatial and temporal variations driven by seasonal water access [23]. The intensive agricultural system in most African countries (especially Egypt) has led to the deterioration of soil properties [24,25].

As a result, several reports indicate that more than 30% of Egypt’s irrigated areas are saline [26]. Egypt has lost approximately 476 hectares (1.9 million acres) in the northern and eastern parts of the Delta due to soil degradation. Urban expansion, soil salinization, pollutants, soil nutrient depletion, soil compaction, land erosion, and dune formation contribute to the destruction of approximately 30,000 acres of prime agricultural land annually [27]. Additionally, the farm sector is estimated to incur annual losses of US$27.3 billion due to agricultural damage caused by saline soils [28]. Many studies in Egypt have presented different solutions to the problem of soil salinity, such as adding compost and plant growth-promoting rhizobacteria to the soil to improve soil properties and reduce the effects of salinity [29,30], compost, elemental sulfur, and sulfur nanoparticles [31], gypsum, compost, and selenium [32], magnetic iron, humic acid and Uni-sal [33], and humic acid, Uni-sal [34].

In this context, several studies have focused on assessing plant diversity in Egypt, such as those by Abd EL-Ghani & Ahmed [35] and El-Zeiny et al. [36], to evaluate and analyze vegetation cover and population centers along the Western Mediterranean Desert. The study by [37] assessed the biodiversity of wild plant habitats and their associated environmental adaptations. In addition, the study of [38], on plant diversity in Fayoum Governorate, [39], on plant diversity in Belbies center, Al-Sharkia governorate, [40,41], on plant diversity in South Western Sinai, Egypt, and [42], on plant diversity in Dakahlia Governorate, and the assessment of vegetation cover and the impact of environmental changes on it. and the Western Desert Oases [43,44], the Nile Delta and Valley [45,46], the Saharan oases [47,48], and reclaimed lands [49], studies in Beni-Suef [50], El-Menoufia [51], and Kharga Oasis [52] further document the interplay between cultivated and wild flora in these arid agroecosystems. This study aimed to quantitatively characterize vegetation composition and edaphic parameters in some salinized agroecosystems of Egypt and investigated the correlation between plant assemblages and soil properties using multivariate analysis.

## Materials and methods

### Floristic Composition

**Fig 1:**
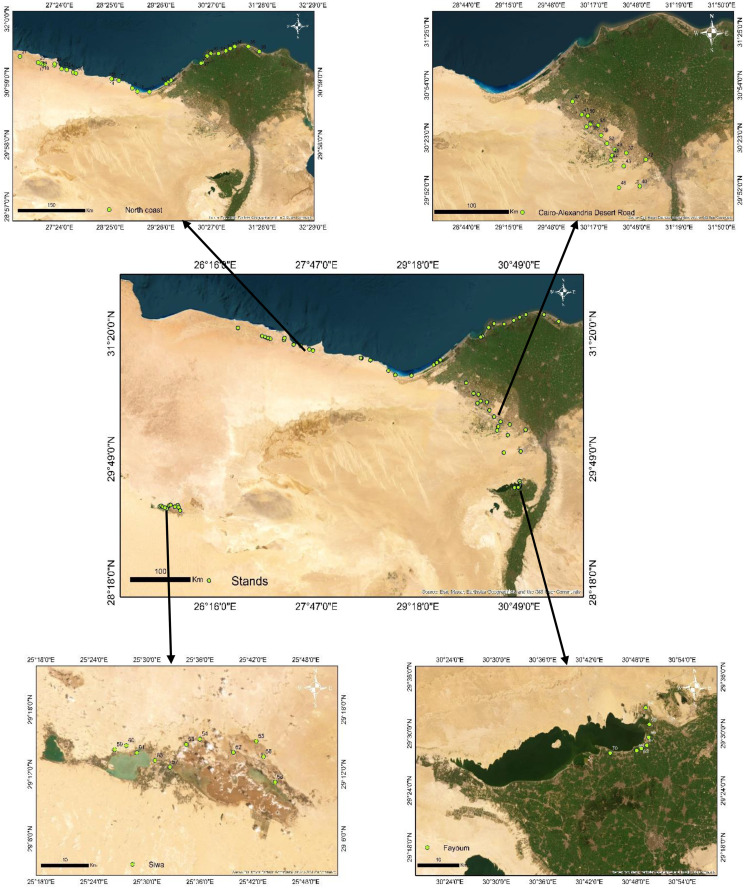
Location map of the 70 stands in agricultural lands affected by salinity in Egypt. Created by the ArcGIS program version 10.5.

To evaluate floristic composition and vegetation cover in some salinized agroecosystems of Egypt, 70 stands were systematically sampled during spring 2022 within each stand, four quadrats (10 m × 10 m; 100 m² per quadrat) were established (Fig 1). The importance value (IV) of each plant species was calculated as a composite index incorporating relative density, relative abundance, relative frequency, and relative cover [53]. Plant species recorded across all stands were taxonomically verified according to [54–58], with updated nomenclature cross-referenced against the Plants of the World Online database (https://powo.science.kew.org/). Species were categorized by life span (annual, biennial, perennial) following Boulos [54–58]. Life form classification (e.g., therophytes, hemicryptophytes) adhered to Raunkiaer’s system [59], while floristic categories (e.g., cosmopolitan, Mediterranean) were assigned based on biogeographic criteria defined by [60,61].

### Soil analyses

Soil physicochemical properties were characterized by assessing 18 parameters: particle size distribution (sand, silt, clay), pH, electrical conductivity (EC), total dissolved salts (TDS), organic matter content, saturation percentage, sodium adsorption ratio (SAR), and calcium (Ca²⁺), magnesium (Mg²⁺), sodium (Na⁺), potassium (K⁺), chloride (Cl⁻), bicarbonate (HCO₃⁻), calcium carbonate (CaCO_3_%), and sulfate (SO₄²⁻). Four subsamples per stand were homogenized to form composite samples for analysis. Particle size fractions were determined using the pipette method [62]. Soil pH, EC, and TDS were measured with a calibrated ADWA^®^ AD8000 series portable meter (Szeged, Hungary). Calcium and magnesium concentrations were quantified via ethylenediaminetetraacetic acid (EDTA) titration [63], whereas sodium and potassium levels were analysed by flame photometry at emission wavelengths of 589 nm (Na⁺) and 767 nm (K⁺) [62]. Chloride and bicarbonate concentrations were assessed using argentometric and acid-base titration [64], while sulfate content was measured turbidimetrically via barium sulfate precipitation [62]. Organic matter was oxidized using the Walkley-Black wet combustion method [65]. SAR was derived from the molar ratio of Na⁺ to (Ca²⁺ + Mg²⁺) in soil solution [66]. Calcium carbonate was determined according to Estefan *et al*. [62] by the titration method. Saturation percentage was determined gravimetrically following standardized protocols [67]. Available N, P, and K were determined in soil samples according to Page et al. [63], Jackson [64], and Black [68], respectively.

### Statistical analysis

Descriptive statistical measures (range, minimum, maximum, mean, standard deviation) were computed for soil physicochemical parameters using SigmaPlot software (version 12.5). Detrended Correspondence Analysis (DCA), a multivariate ordination technique, was implemented in PC-ORD version 5 to examine associations between diagnostic plant species and edaphic gradients influencing stand distribution patterns.

## Results and discussion

### Floristic composition

**Fig 2:**
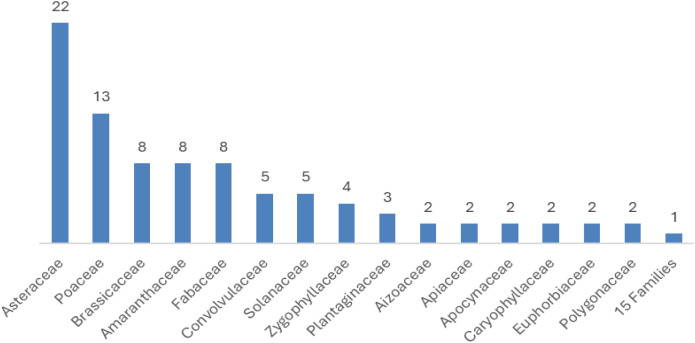
Graphical representation of angiosperm families according to the number of species.

A total of 103 plant taxa representing 15 families were documented in salinity-affected agricultural lands of Egypt (Tables 1, 3, and Fig 2). Asteraceae and Poaceae dominated the floristic composition, contributing 22 species (21.4%) and 13 species (12.6%). Brassicaceae, Amaranthaceae, and Fabaceae each comprised 8 species (7.8%), while Convolvulaceae and Solanaceae contributed 5 species each (4.9%). Zygophyllaceae was represented by 4 species (3.9%), and Plantaginaceae by 3 species (2.9%). Aizoaceae, Apiaceae, Apocynaceae, Caryophyllaceae, Euphorbiaceae, and Polygonaceae each included 2 species (1.9%). Fifteen families were represented by a single species (Table 1).

**Table 1:** List of species recorded in the agroecosystem of agricultural lands affected by salinity in 2022. Species were assigned to their respective families, and life span; Annual = Ann, Biennial = Bie, Perennial=Per., life forms; Therophyte = Th, Hemicryptophyte = Hem, Chamaephyte = Cha, Phanerophyte = Ph, Geophyte = Geo, Helophyte = Hel, Phyto–geographical affinities; COSM = Cosmopolitan, ER–SR = Euro–Siberian, IR–TR = Irano–Turanian, ME = Mediterranean, PAL = Paleotropical, PAN = Pantropical, S–Z = Sudano–Zambesian. Arabic names are listed. Families are arranged alphabetically, with genera and species listed alphabetically within each family. A = North Coast, B = Desert Road Cairo-Alexandria, C = Siwa, and D = Fayoum.

Species	A	B	C	C	Family	Life form	Life span	Floristic categories	Arabic name	Abbreviation
*Achillea fragrantissima* (Forssk.) Sch.Bip.	1	0	0	0	Asteraceae	Cha.	Per.	IR-TR + SA	قيصوم	Ach fra
*Alhagi graecorum* Boiss.	0	0	1	1	Fabaceae	Hem.	Per.	PAL	عاقول	Alh gra
*Amaranthus caudatus* L.	0	1	0	0	Amaranthaceae	Th.	Ann.	COSM	عرف الديك	Ama cau
*Anagallis arvensis* L. (*Lysimachia arvensis* (L.) U. Manns & Anderb.)	1	0	1	1	Primulaceae	Th.	Ann.	ME + IR-TR + ER-SR	ذيل الفار - ياداب	Ana arv
*Arthrocnemum macrostachyum* (Moric.) K. Koch (*Arthrocaulon macrostachyum* (Moric.)	1	0	1	1	Amaranthaceae	Cha.	Per.	ME + SA	شنان	Art mac
*Astragalus incanus* L.	1	0	0	0	Fabaceae	Cha.	Per.	IR-TR + SA	العشبة السنامية	Ast inc
*Astragalus spinosus* (Forssk.) Muschl.	0	1	0	0	Fabaceae	Cha.	Per.	IR-TR + SA	قتاد	Ast spi
*Atriplex halimus* L.	1	1	0	0	Amaranthaceae	Ph.	Per.	IR-TR + SA	شجيرة الملح	Atr hal
*Avena fatua* L.	1	1	0	0	Poaceae	Th.	Ann.	COSM	زمير - خافور	Ave fat
*Bassia indica* (Wight) A.J. Scott	1	0	0	0	Amaranthaceae	Th.	Ann.	IR-TR + SA	العذام	Bas ind
*Beta vulgaris* L.	0	0	0	0	Amaranthaceae	Th.	Ann.	ME + IR-TR + ER-SR	البنجر	Bet vul
*Brassica tournefortii* Gouan (*Coincya tournefortii* (Gouan))	1	0	0	0	Brassicaceae	Th.	Ann.	COSM	العسلوز	Bra tou
*Bromus diandrus* Roth	0	1	0	0	Poaceae	Th.	Ann.	ME + IR-TR + S-Z	الشويعرة الضخمة	Bro dia
*Calendula officinalis* L.	1	0	1	1	Asteraceae	Th.	Ann.	ME + IR-TR	زهرة اذريون	Cal off
*Calotropis procera* (Aiton) Dryand.	1	0	0	0	Apocynaceae	Th.	Ann.	SA + SZ	القطف - شجرة الحليب	Cal pro
*Capsella bursa-pastoris* (L.) Medik.	0	1	0	0	Brassicaceae	Ph.	Per.	COSM	كيس الراعى	Cap bur
*Carduus getulus* Pomel	1	1	0	0	Asteraceae	Th.	Ann.	IR-TR + SA	الشوك - الخرشوف البري	Car get
*Centaurea aegyptiaca* L.	1	1	0	0	Asteraceae	Th.	Ann.	SA	يمرار	Cen aeg
*Centaurea calcitrapa* L.	1	0	0	0	Asteraceae	Cha.	Bie.	ME + ER-SR	مرار	Cen cal
*Chenopodium murale* (L.) (*Chenopodiastrum murale* (L.) S. Fuentes, Uotila & Borsch)	1	0	0	0	Amaranthaceae	Cha.	Bie.	COSM	لسان الطير -أبوعفين	Che mur
*Cichorium endivia* L.	1	1	1	1	Asteraceae	Th.	Ann.	ME + IR-TR	شيكوريا	Cic end
*Convolvulus althaeoides* L.	1	0	1	1	Convolvulaceae	Th.	Ann.	ME + IR-TR	العكار	Con alt
*Convolvulus hystrix* Vahl	1	1	0	0	Convolvulaceae	Geo.	Per.	ME + IR-TR	الرباط الشوكي	Con hys
*Convolvulus arvensis* L. (*Ipomoea maurandioides* Meisn.)	1	0	0	0	Convolvulaceae	Geo.	Per.	PAL	عليق	Con arv
*Conyza bonariensis* (L.) Cronquist (*Erigeron bonariensis* L.)	1	1	1	1	Asteraceae	Th.	Ann.	ME	حشيش الجبل	Con bon
*Cornulaca monacantha* Delile	1	1	0	0	Amaranthaceae	Cha.	Per.	IR-TR	حاد - شوك الذيب	Cor mon
*Coronopus squamatus* (*Lepidium coronopus* (L.) Al-Shehbaz)	0	1	0	0	Brassicaceae	Th.	Ann.	ME + SA	الكرنب البري	Cor squ
*Cressa cretica* L.	1	1	1	1	Convolvulaceae	Hem.	Per.	PAL	مليح - ندوة	Cre cre
*Cynanchum acutum* L.	0	0	1	1	Apocynaceae	Ph.	Per.	ME + IR-TR + ER-SR	عليق - لبين	Cyn acu
*Cynodon dactylon* (L.) Pers.	1	1	1	1	Poaceae	Geo.	Per	PAN	نجيل	Cyn dac
*Cyperus rotundus* L.	1	1	1	1	Cyperaceae	Geo.	Per.	PAN	بربيط	Cyp rot
*Desmostachya bipinnata (*L.) Stapf	1	0	0	0	Poaceae	Hem.	Per.	SA + S-Z	حلفا	Des bip
*Deverra tortuosa* (Desf.) DC.	0	1	0	0	Apiaceae	Cha.	Per.	SA	شبت الجبل	Dev tor
*Diplotaxis muralis* (L.) DC.	1	0	0	0	Brassicaceae	Th.	Ann.	ME + IR-TR	خفج جداري	Dip mur
*Echinochloa crus-galli* (L.) P. Beauv.	1	0	0	0	Poaceae	Th.	Ann.	ME + IR-TR	دنيبة	Ech cru
*Echinops spinosus* (*Echinops spinosissimus* subsp. *Spinosissimus*)	0	0	0	0	Asteraceae	Hem.	Per.	ME + S-Z	قتاد	Ech spi
*Emex spinosa* (L.) (*Rumex spinosus* L.)	0	1	0	0	Polygonaceae	Th.	Ann.	ME + IR-TR	حمباز شوكي	Eme spi
*Erodium laciniatum* (Cav.) Willd.	1	1	0	0	Geraniaceae	Th.	Ann.	SA	دهمية	Ero lac
*Eruca sativa* Mill.	1	1	0	0	Brassicaceae	Th.	Ann.	ME + IR-TR + ER-SR + SA	جرجير	Eru sat
*Euphorbia helioscopia* L.	1	1	1	1	Euphorbiaceae	Th.	Ann.	COSM	سعده	Eup hel
*Euphorbia peplus* L.	1	0	0	0	Euphorbiaceae	Th.	Ann.	COSM	ودينه – زغلنته	Eup pep
*Fagonia arabica* L. (*Zygophyllum arabicum* (L.) Christenh. & Byng)	1	1	0	0	Zygophyllaceae	Cha.	Per.	ME + SA	عشبة الفاغونيا	Fag ara
*Foeniculum vulgare* Mill.	1	1	0	0	Apiaceae	Hem.	Per.	ME + IR-TR	الشمر	Foe vul
*Glebionis coronaria* (L.) Cass. ex Spach	0	1	0	0	Asteraceae	Th.	Ann.	ME	اقحوان	Gle cor
*Glycyrrhiza glabra* L.	1	0	0	0	Fabaceae	Ph.	Per.	ME	العرقسوس	Gly gla
*Haloxylon salicornicum* (Moq.) Bunge ex Boiss.	1	0	1	1	Plantaginaceae	Cha.	Per.	S-Z	رمث	Hal sal
*Heliotropium ovalifolium* forssk. (*Euploca ovalifolia* (Forssk.) Diane & Hilger)	0	1	0	0	Boraginaceae	Th.	Ann.	ME + IR-TR	غبيرة	Hel ova
*Hordeum marinum* Huds.	0	0	1	1	Poaceae	Th.	Ann.	ME + IR-TR	الشعير البحري	Hor mar
*Imperata cylindrica* (L.) Raeusch.	0	1	0	0	Poaceae	Hem.	Per.	ME + S-Z	الحلفا	Imp cyl
*Ipomoea cairica* (L.) Sweet	0	1	1	1	Convolvulaceae	Hem.	Per.	PAL	ست الحسن	Ipo cai
*Juncus rigidus* Desf.	1	0	0	0	Juncaceae	Hem.	Per.	ME + IR-TR + SA	سمار حصر- سمار مر	Jun rig
*Launaea nudicaulis* (L.) Hook.f.	1	0	0	0	Asteraceae	Hem.	Per.	IR-TR	حوذان	Lau nud
*Lolium perenne* L.	1	1	0	0	Poaceae	Hem.	Per.	ME + IR-TR + ER-SR	نصيل-حشيش الفرس	Lol per
*Lotus glaber* Mill. (*Lotus tenuis* Waldst. & Kit. ex Willd.)	0	0	1	1	Fabaceae	Hem.	Per.	ME + IR-TR + ER-SR	قرن الغزال	Lot gla
*Lycium europaeum* L.	1	0	1	1	Solanaceae	Ph.	Per.	ME + SA + S-Z	الباذنجان البري	Lyc eur
*Malva parviflora* L.	1	1	0	0	Malvaceae	Th.	Ann.	ME + IR-TR	الخبيزة	Mal par
*Matthiola livida* Kralik ex Pomel (*Matthiola kralikii* Pomel)	1	1	1	1	Brassicaceae	Th.	Ann.	ME	يسنيو - شقارة - شمشم	Mat sin
*Medicago sativa* L.	1	0	0	0	Fabaceae	Hem.	Per.	ME + IR-TR + ER-SR	برسيم حجازى	Med sat
*Melilotus indicus* (L.) All.	1	1	0	0	Fabaceae	Th.	Ann.	PAL	حندقوق مر	Mel ind
*Mentha longifolia* (L.) L.	1	1	1	1	Lamiaceae	Hem.	Per.	ME + IR-TR + ER-SR	فليه – حبق الميه	Men lon
*Mesembryanthemum crystallinum* L.	1	0	0	0	Aizoaceae	Th.	Ann.	ME + IR-TR + SA	نبات الثلج	Mes cry
*Mesembryanthemum nodiflorum* L.	1	0	0	0	Aizoaceae	Th.	Ann.	ME + ER-SR + SA	غسول	Mes nod
*Nicotiana glauca* Graham	1	0	0	0	Solanaceae	Ph.	Per	ME + IR-TR + SA	التبغ البري	Nic gla
*Onopordum alexandrinum* Boiss.	1	0	0	0	Asteraceae	Hem.	Per.	ME + IR-TR + SA	الخرشوف البري	Ono ale
*Oxalis corniculata* L.	1	1	0	0	Oxalidaceae	Th.	Ann	COSM	حمد	Oxa cor
*Pancratium maritimum* L.	1	0	0	0	Amaryllidaceae	Th.	Per.	ME + IR-TR + SA	زنبق البحر	Pan mar
*Phoenix dactylifera* L.	1	1	0	0	Arecaceae	Geo.	Per.	SA + S-Z	نخيل البلح	Pho dac
*Phragmites australis* (Cav.) Trin. ex Steud.	1	0	1	1	Poaceae	Ph.	Per.	PAL	بوص-غاب	Phr aus
*Plantago major* L.	1	1	1	1	Plantaginaceae	Geo.	Per.	ME + IR-TR	مصاصة	Pla maj
*Plantago squarrosa* Murray	1	0	0	0	Plantaginaceae	Hem.	Ann.	ME + IR-TR + SA	لسان الحمل السهمي	Pla squ
*Pluchea dioscoridis* (L.) DC.	1	0	0	0	Asteraceae	Th.	Per.	SA + S-Z	برنوف	Plu dio
*Polypogon monspeliensis* (L.) Desf.	1	0	1	1	Poaceae	Ph.	Ann.	COSM	ذيل القط	Pol mon
*Pulicaria undulata* (L.) C.A. Mey.	1	0	1	1	Asteraceae	Th.	Per.	SA + S-Z	جثجاث	Pul und
*Reichardia tingitana* (L.) Roth	1	1	0	0	Asteraceae	Cha.	Ann.	ME + IR-TR + SA	مرار	Rei tin
*Reseda pruinosa* Delile	1	1	0	0	Resedaceae	Th.	Ann.	ME + IR-TR + SA	دنبان	Res pru
*Rumex dentatus* L.	1	0	0	0	Polygonaceae	Th.	Ann.	ME + IR-TR + SA	خلاله	Rum den
*Rumex vesicarius* L.	0	0	0	0	Asteraceae	Th.	Ann.	ME + SA + S-Z	حماض	Rum ves
*Senecio vulgaris* L.	1	0	0	0	Asteraceae	Th.	Ann.	ME + IR-TR + SA	مرار	Sen vul
*Senecio glaucus* L.	1	1	0	0	Asteraceae	Th.	Ann.	ME + IR-TR + SA	قريص -أم لونين	Sen gla
*Setaria viridis* (L.) P. Beauv.	1	1	1	1	Poaceae	Th.	Ann.	ME + IR-TR + SA	ذيل الفار- ياداب	Set vir
*Silybum marianum* (L.) Gaertn.	1	0	0	0	Asteraceae	Th.	Ann.	ME + IR-TR	شوك سناري - شك الجمل	Sil mar
*Sisymbrium irio* L.	1	1	0	0	Brassicaceae	Th.	Ann.	ME + IR-TR	فجل الجمل	Sis iri
*Solanum nigrum* L.	1	1	0	0	Solanaceae	Th.	Ann.	ME + IR-TR + ER-SR	عنب الذيب	Sol nig
*Solanum villosum* Mill.	1	0	0	0	Solanaceae	Th.	Ann.	ME + IR-TR + SA	الباذنجان الاحمر	Sol vil
*Sonchus maritimus* L.	0	1	0	0	Asteraceae	Hem.	Per.	ME + IR-TR	سيفون	Son mar
*Sonchus oleraceus* L.	0	0	0	0	Asteraceae	Th.	Ann.	COSM	جعضيض– جلاوين	Son ole
*Sorghum halepense* (L.) Pers.	1	1	1	1	Poaceae	Cha.	Per.	PAL	ذرة صيفى – ذرى عويجة	Sor hal
*Spergularia marina* (L.) Besser	1	0	0	0	Caryophyllaceae	Hem.	Ann.	ME + IR-TR + ER-SR	ابوغلام	Spe mar
*Suaeda pruinosa* Lange	1	1	1	1	Amaranthaceae	Cha.	Per.	ME + IR-TR + SA	عشبة البحر	Sua pru
*Tamarix nilotica* (Ehrenb.) Bunge	1	1	0	0	Tamaricaceae	Ph.	Per.	SA + S-Z	عبل – طرفة	Tam nil
*Thymelaea hirsuta* (L.) Endl.	1	0	1	1	Thymelaeaceae	Ph.	Per.	ME + IR-TR + SA	المثنان الشوكي	Thy hir
*Tribulus terrestris* L.	1	1	0	0	Zygophyllaceae	Th.	Ann.	IR-TR + ER-SR + S-Z	ضريس	Tri ter
*Trigonella arabica* Delile	1	0	0	0	Fabaceae	Th.	Ann.	ME + IR-TR + SA	الحمص العربي	Tri ara
*Typha domingensis* Pers.	1	1	0	0	Typhaceae	Hel.	Per.	PAN	ديس- بردي - بوط	Typ dom
*Urospermum picroides* (L.) Scop. ex F.W.Schmidt	1	0	0	0	Asteraceae	Th.	Ann.	ME + IR-TR	بسيخ	Uro pic
*Urtica urens* L.	1	1	0	0	Urticaceae	Th.	Ann.	ME + ER-SR	حريق - قريص	Urt ure
*Vaccaria pyramidata* Medik. (*Gypsophila vaccaria* (L.) Sm.)	1	0	0	0	Caryophyllaceae	Th	Ann	ME + ER-SR	فول العرب	Tam nil
*Withania somnifera* (L.) Dunal	1	0	0	0	Solanaceae	Cha.	Per.	SA + S-Z	سم الفراخ	Wit som
*Xanthium spinosum* L.	1	0	0	0	Asteraceae	Th.	Ann.	PAL	شبيط	Xan spi
*Zilla spinosa* (L.) Prantl	1	0	0	0	Brassicaceae	Cha.	Per.	ME + IR-TR + ER-SR + SA	سلة	Zil spi
*Zygophyllum album* L.f.	1	0	0	0	Zygophyllaceae	Cha.	Per.	ME + IR-TR + SA + S-Z	رطريط -بوال	Zyg alb
*Zygophyllum coccineum* L.	0	1	0	0	Zygophyllaceae	Cha.	Per.	SA + S-Z	قلم – بلبال	Zyg coc

The results of this study are entirely consistent with many previous studies conducted in similar areas in Egypt, such as Al-Sherif *et al.* [38], aiming to determine the composition and distribution of grass plants in different habitats in Fayoum. The results recorded 175 vascular plant species belonging to 124 genera and 35 families, distributed over eight habitats. The richest families were Poaceae, Asteraceae, and Fabaceae. Ellmouni *et al.* [69] studied the vegetation composition of trees and grass in four public parks in the Fayoum area (Fayoum University Gardens - FUGs, Fayoum International Farms Park – FPIG, Fayoum Governorate Club – FGC, and Fayoum Zoo – FZ). Two hundred and sixteen species and one hundred and fifty-one genera representing 58 plant families were identified. The Asteraceae, Moraceae, and Fabaceae families are the richest families in the floristic composition of the area. Bedair [70] studied the flora of weeds in the agricultural system in the Siwa Oasis and found that the Asteraceae and Poaceae plant families were the most represented among the plant families. Asteraceae was also the most represented family in many areas in Egypt, such as Wadi Hagul [71], Wadi Hof Eastern Desert, Egypt [72]. Poaceae was the most represented, followed by the Asteraceae, in a study of the agroecosystem in El-Menoufia Governorate, Nile Delta, Egypt [73]. This is partially consistent with the study by Shaltout *et al.* [74], evaluating the floristic composition associated with high-voltage pylon platforms constructed to support power line towers in the Northern Nile Delta. The largest families recorded on the Mediterranean coast of the Northern Nile Delta were Asteraceae (16 species), Poaceae (15 species), Chenopodiaceae (12 species), and Fabaceae (7 species). Ten aliens (10.7%) out of 84 species were recorded. Asteraceae make up the bulk of the floristic composition in Egypt. It is represented by 98 genera and 234 species [75]. Asteraceae is recognized for encompassing a notable proportion of species adapted to saline environments and xerophytic conditions [76]. Poaceae is widely recognized for its ability to tolerate drought, salinity, freezing temperatures, and other abiotic stressors [77].

In addition to the 103 wild taxa, 11 cultivated species from 10 families were recorded in these salinized agroecosystems (Table 2).

**Table 2:** A list of the cultivated species recorded in agricultural lands affected by salinity in Egypt and their families, genera, and species is in alphabetical order within their respective families.

Species	Family	Arabic name	Abbreviation
*Citrus sinensis* (L.) Osbeck.	Rutaceae	البرتقال	Cit sin
*Eruca sativa* Mill.	Brassicaceae	جرجير	Eru sat
*Ficus carica* L.	Moraceae	التين	Fic car
*Hordeum vulgare* L.	Poaceae	الشعير	Hor vul
*Mangifera* sp	Anacardiaceae	مانجو	
*Olea europaea* L.	Oleaceae	الزيتون	Ole eur
*Phoenix dactylifera* L.	Arecaceae		Pho dac
*Psidium guajava* L.	Myrtaceae	الجوافة	Psi gua
*Triticum aestivum* L.	Poaceae	قمح	Tri aes
*Vicia faba* L.	Fabaceae	فول	Vic fab
*Vitis vinifera* L.	Vitaceae	عنب	Vit vin

**Table 3:** Total recorded families and number of species within each family, and their percentage within the four studied locations.

Family	NO.	%
Asteraceae	22	21.4
Poaceae	13	12.6
Brassicaceae	8	7.8
Amaranthaceae	8	7.8
Fabaceae	8	7.8
Convolvulaceae	5	4.9
Solanaceae	5	4.9
Zygophyllaceae	4	3.9
Plantaginaceae	3	2.9
Aizoaceae	2	1.9
Apiaceae	2	1.9
Apocynaceae	2	1.9
Caryophyllaceae	2	1.9
Euphorbiaceae	2	1.9
Polygonaceae	2	1.9
15 Families	1	1.0

**Fig 3:**
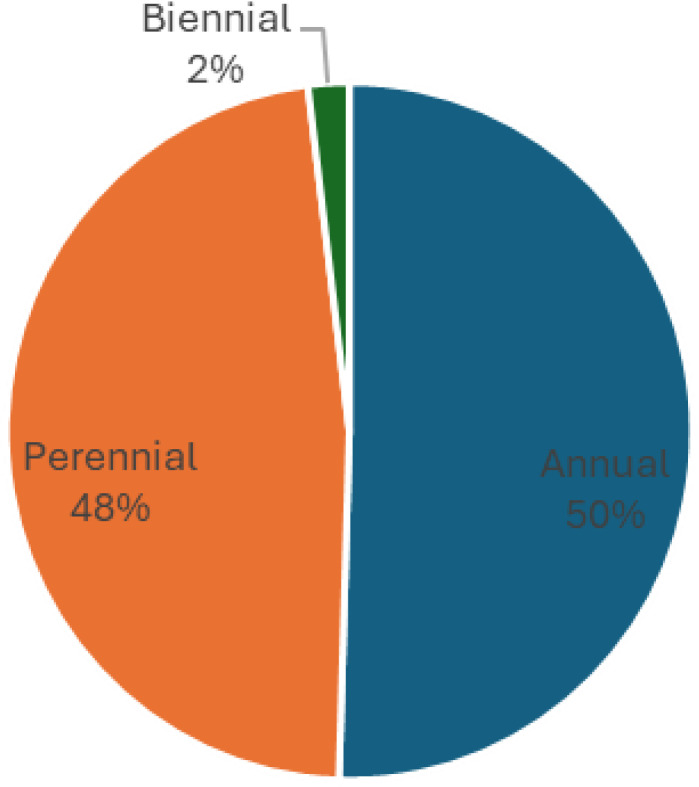
Life span of plant species in agricultural lands affected by salinity in Egypt.

Regarding life span, annual species predominated, accounting for 52 taxa (50.5% of the total species), followed by perennials with 49 taxa (47.6%). Only two biennial species, *Centaurea aegyptiaca* and *Centaurea calcitrapa*, were observed (Fig 3). This is consistent with the study of Shaltout *et al.* [74], where it was recorded in Mediterranean in North Nile Delta, 47 annuals (56.0%), 35 are perennials (41.6%), belonging to 23 families. and study There is agreement with the study of EL-Shennawy *et al.* [39], Which was conducted to conduct a botanical survey and phytogeographical study for the Belbeis Center in Sharkia Governorate, the results showed in the various habitats studied in the study region, 88 species, including annuals,33 perennials, 3 biannuals, were connected to the studied families.

These results are consistent with the results of Abd El-Ghani *et al.* [78], a study on habitat diversity and floristic analysis of Wadi El-Natrun Depression, Western Desert, Egypt, which showed that: Habitat diversity and floristic analysis of Wadi El-Natrun Depression, Western Desert, Egypt. A total of 142 species of vascular plants belonged to 108 genera in 35 families; they consisted of 21 trees and shrubs (14.79%), 42 perennial herbs (29.58%), and 79 annuals (55.63%).

**Fig 4:**
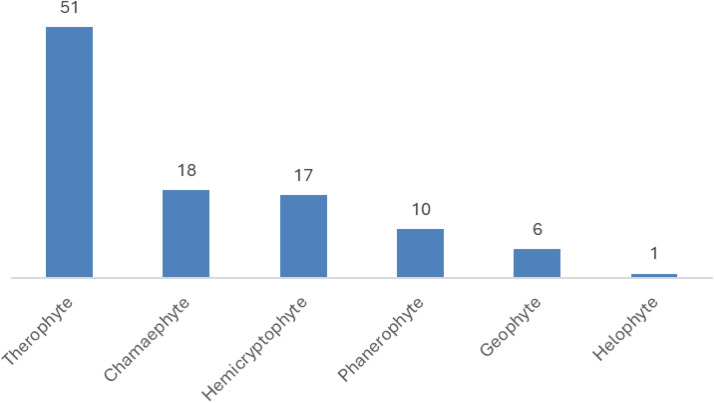
Life forms of plant species in agricultural lands affected by salinity in Egypt.

Six life forms were identified across the studied stands: therophytes (51 species, 49.5%), chamaephytes (18 species, 17.5%), hemicryptophytes (17 species, 16.5%), phanerophytes (10 species, 9.7%), geophytes (6 species, 5.8%), and helophytes (1 species, 1%; *Typha domingensis*) Fig 4. Plant life forms have evolved through adaptations to environmental and climatic conditions [79]. In desert ecosystems, plant life form patterns are primarily associated with rainfall, as well as topography and landform [80,81]. In the studied area, therophytes represented the most prevalent life form at 51% of recorded species, followed by Chamaephytes at 18%. Other life forms each constituted less than 31%. Most annual species were predominantly spring or cool-season specialists. Notably, elevated proportions of Fabaceae and therophytes in regional flora may serve as an indicator of ecosystem disturbance in Mediterranean regions [49]. While in the study of Ellmouni *et al.* [69], Phanerophytes (46.5%) were the most common life form, followed by Hemicryptophytes (38%). in the study of Al-Sherif *et al.* [38], it was the therophytes that were the dominant life form, while Phanerophytes were the smallest group in this study, 5%.

**Fig 5:**
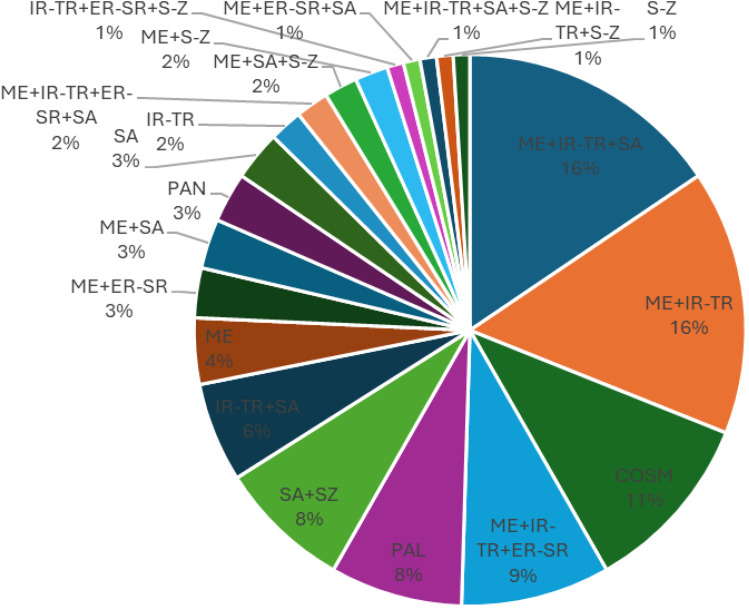
Floristic categories of recorded species in agricultural lands affected by salinity in Egypt.

Phytogeographical analysis classified the recorded species into four groups: cosmopolitan (22 species, 21.4%), monoregional (10 species, 9.7%), biregional (38 species, 36.9%), and pleuriregional (33 species, 32%) (Fig 5). This is partially consistent with the study by El-Saied *et al.* [44], who found that uncultivated species in Siwa Oasis consisted mostly of monoregional (28), bioregional (27), and pantropical (20) species with regional affinities spanning North Africa, the Mediterranean, and Central Asia. Additional phytogeographic categories included palaeotropical (14 species), cosmopolitan (9 species), and pantropical (4 species). Furthermore, Ellmouni *et al.* [69] found four basic phytogeographic groups: cosmopolitan, biregional, panregional, and monoregional. The highest rates of participation were observed in the regional and monoregional categories, at 21% and 53%, respectively. Twenty-seven species (13%) were found to be native to the Sahara-Arabia region. Meanwhile, in the study of Abd El-Ghani & Abdel–Khalik [49], which was conducted to analyze the floristic composition around wells and springs in five oases (Siwa, Bahariya, Farafra, Dakhla, and Kharga) in the Egyptian Western Desert in terms of habitat were recorded as the most common families in the oases’ agroecosystems. Poaceae, Asteraceae, and Fabaceae families were found to be the most common families.

### Soil analysis

The results of descriptive statistics for soil samples collected from the studied stands are summarized in Table 4.

**Table 4:** Descriptive summary statistics for the soil analysis results of the 70 soil samples collected during spring from agricultural lands affected by salinity in Egypt.

Soil parameters	Mean±Standard error	Range (Max-Min)	Standard deviation	Median
pH	8.01 ± 0.0272	1.2(8.6-7.4)	0.227	8
EC dS/m	46.424 ± 10.68	287.03(287.5-0.47)	89.354	2.925
Ca ^+ 2^	31.386 ± 5.188	240.15(242.42-2.27)	43.402	10.225
Mg ^+ 2^	80.313 ± 19.938	765.37(766.67-1.3)	166.814	8.77
Na^+^	453.895 ± 123.013	4699.39(4700-0.61)	1029.199	10.785
K^+^	8.862 ± 2.613	126.69(126.69−0)	21.863	0.865
HCO3^-^	3.542 ± 0.238	11.56(12.74-1.18)	1.991	3.3
Cl^–^	496.761 ± 128.384	4370.34(4372.88-2.54)	1074.135	12.71
SO_4_ ^− −^	74.15 ± 15.077	571.6(572.64-1.04)	126.141	13.48
SAR	39.508 ± 13.115	800.8(801.16-0.36)	109.727	3.305
T.D.S.	29711.223 ± 6835.083	183698.24(184000-301.76)	57186.404	1873.12
SP	31.129 ± 0.715	25(50−25)	5.978	28
CaCO_3_%	30.188 ± 2.6	74.96(80.4-5.44)	21.752	24.505
Available K^+^	326.485 ± 47.049	1805.35(1811.76-6.41)	393.639	197.56
Available P ^− −^	4.349 ± 0.652	28.99(29.03-0.04)	5.454	2.545
Available N	79.452 ± 2.466	87.25(126-38.75)	20.629	77.5
Organic matter	0.617 ± 0.04	1.5(1.53-0.03)	0.335	0.555
sand %	75.64 ± 1.58	64(96−32)	13.218	78.75
clay %	16.745 ± 0.715	37.75(40.25-2.5)	5.979	16.515
Silt %	7.615 ± 1.144	38.97(39.22-0.25)	9.568	4.005

### A- Physical analyses

#### 1- Saturation percentage (SP).

The highest saturation rate (50%) was recorded in the Fayoum samples (S65, S67). While the lowest saturation rate (25%) was recorded in the North Coast sample (S2), Cairo-Alexandria Desert Road samples (S41, S43, S44, S47, S48, S51), and Siwa Oasis samples (S53, S54, S61) (average 31.128%). The saturation rate ranged between 0 and 25%. In the North Coast, the saturation rate ranged between 25 and 35%, based on the results of Abdelaal *et al.* [82], which ranged between 18 and 54%. On the Cairo-Alexandria Desert Road, the rate ranged between 25 and 43%, Siwa between 25 and 42%. In the study by Elnaggar *et al.* [83], in Siwa Oasis, the rate ranged between 17% and 59%, and in Fayoum between 30% and 50% (Table 4). This indicates that there is a difference between the results. This may be due to soil properties changing over time due to farmers’ reliance on wastewater irrigation and the excessive use of fertilizers and pesticides, which accumulate in the soil and alter their properties, such as the saturation rate, this may be due to soil properties changing over time as a result of some farmers’ reliance on wastewater irrigation and the excessive use of fertilizers and pesticides, which accumulate in the soil and alter its properties, such as the saturation rate.

#### 2- Soil texture.

**Fig 6:**
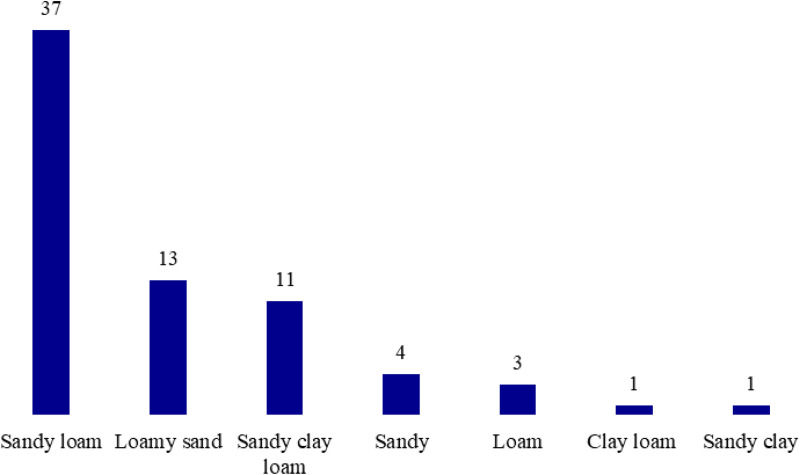
The soil texture types and the percentages of each type in the studied stands.

Soil samples collected from different locations showed a significant variation in texture. In spring, the most common soil type was sandy loam (52.9%), while loamy sand and sandy clay loam soil had similar proportions of 18.6% and 15.7%, respectively. Sandy, loamy, sandy, clay, and clay loam were the most similar soil types, ranging from 1.4% to 5.7%. Sandy soil was the most common on the North Coast, with 61.11% of the samples being sandy. On the Desert Highway, the proportion of sandy loam soil was equal to loamy sand soil, 37.5%. In Siwa, 70% of the samples were sandy loam, and in Fayoum, 42.85% of the samples were sandy clay loam (Table 5 and Fig 6).

**Table 5:** Different soil texture classes recorded within all studied locations and their percentages.

Soil texture	No. of stands	%
Sandy loam	37	52.9
Loamy sand	13	18.6
Sandy clay loam	11	15.7
Sandy	4	5.7
Loam	3	4.3
Clay loam	1	1.4
Sandy clay	1	1.4

#### 3- Hydrogen ion concentration (pH).

In general, the pH values of soil samples collected from 70 different stands ranged from 7.40 in Fayoum to 8.60 in Siwa Oasis. The pH values of soil samples collected from the 70 studied stands were slightly alkaline to alkaline, ranging from 7.70–8.4 on the northern coast. This is partially consistent with the study conducted by Bedair *et al.* [84], which was conducted along the Mediterranean coast to describe plant communities and provide information on their distribution and the soil variables that affect them. The study showed pH values ranging from 9.4 to 7.3 (Table 4). Along the desert highway, pH values ranged from 7.70–8.20. In the Siwa Oasis, pH values ranged from 7.60–8.60. This value is partially consistent with the study by El-Hassanin *et al.* [85], to assessed the potential for improving and expanding cultivated areas within the oasis itself using remote sensing and geographic information systems (GIS) techniques. pH values ranged from 7.8 to 9.5, while pH values in Fayoum ranged from 7.40 to 8.60. This is partially consistent with the study by Abdel-Fattah *et al.* [86], who collected 36 soil samples from agricultural lands near Lake Qarun and found that soil pH values ranged from 7.5 to 8.6, indicating that conditions in the study area were moderately strongly alkaline results of the current study are also consistent with the results of Zaid *et al.* [87], and El-Zeiny & Effat, [88] who collected soil samples from 40 stands to study the effect of wastewater irrigation on soil properties (7.83–8.31). and soil pH varied from 8.10–8.97 (about 8.41 on average).

### B- Chemical analysis

#### 1-Electrical conductivity (EC).

The electrical conductivity displayed showed a similar trend to the total dissolved solids (TDS) values, with the highest value (28.5 ds/m) recorded on the North Coast, while the lowest value (0.62 ds/m) was recorded, ranging between 0.47–279.45 on the Cairo-Alexandria Desert Road. In Siwa Oasis, the value ranged between 10.04–287.5 ds/m, and in Fayoum, values ranging between 5.15–277.15 ds/m were recorded, with a significant difference between the study areas (Table 4). Soil electrical conductivity is of critical importance, reflecting the content of water-soluble salts, particularly sodium, potassium, calcium, and magnesium, but also including chlorides, sulfates, and carbonates. These can severely impact plant growth and land use and increase soil erosion [89,90]. Richards [91] distinguished between five classes of soil salinity, from non-saline to highly saline. Accordingly, the soils of the study area were classified as shown in Table 6.

**Table 6:** Soil classification of the study area according to Richard’s classification.

location	EC (dS/m)	Soil salinity classification	Impact on agriculture
North Coast	0.62–28.5	Non-saline- Very strongly saline	No noticeable effect on plant growth- Not suitable for cultivation except for tolerant crops
Cairo-Alexandria Desert Road.	0.47-279.45	Non-saline- Very strongly saline	No noticeable effect on plant growth- Not suitable for cultivation except for tolerant crops
Siwa Oasis	10.04-287.5	Strongly saline- Very strongly saline	Reduces yields of most crops- Not suitable for cultivation except for tolerant crops
Fayoum	5.15-277.15	Moderately saline- Very strongly saline	Affects most sensitive crops- Not suitable for cultivation except for tolerant crops

The electrical conductivity of the North Coast soil ranged from non-saline (0.62 dS/m) to highly saline (28.5 dS/m). These results are partially consistent with the results of Hagage *et al.* [92], which showed that 30–40% of the Nile Delta soil was affected by salt, while up to 64% of the northeast Delta samples were highly saline or very saline, and electrical conductivity values were typically >20 dS/m. Along the Cairo–Alexandria Desert Road, EC varies widely from 0.47 dS/m (non-saline) to 279.45 dS/m (very high). A new study justified this wide variation: Mohamed *et al.* [93], reported soil EC levels in reclaimed desert soils from a minimum of 0.9 dS/m to as high as 236 dS/m, with an average EC of 21.5 dS/m, with significant spatial heterogeneity and areas of extreme salinity. Similarly, in Wadi El-Madamude, East Luxor, Upper Egypt region, Elwan & Barseem, [94] have also reported EC values from 0.8–245 dS/m, and they have stressed the impact of marginal water irrigation and soil salinity buildup on soil characteristics. In the Siwa Oasis, the oasis soils ranged from very saline (10.04 ds/m) to very saline (287.5 ds/m). This is partially consistent with a study by Elnaggar *et al.* [83]. Most soils were very saline, based on conductivity values ranging from 4.25 to 427 ds/m. Meanwhile, the soil in Fayoum ranged from moderately saline (5.15 ds/m) to highly saline (277.15 ds/m). The results of the current study are partially consistent with the study by El-Zeiny & Effat [88], which showed that the electrical conductivity value ranged from 0.04 to 9.59 ds/m.

#### 2- Total Soluble Salts (T.D.S.).

The results in Table 4 reveal significant variation in total dissolved salt concentrations, ranging from 301.76 mg/l in the Desert Road sample (S48) to 184,000 mg/l in the Siwa Oasis sample (S63) (with an average of 62,082.43 mg/l). There was significant variation between the study areas. The coastal area is characterized by saline soils, with total dissolved salt concentrations ranging from 404.80 to 18240 mg/l. These results demonstrate significant variation in soil salinity, which is rich in salt and has a high salinity level. These soils support salt-tolerant plants.

The region is exposed to salinization processes due to several factors, such as the rising level of saltwater seeping from Lake Manzala. In addition, the high temperature in the summer accelerates the formation of thin layers of salt on the surface soil [95]. In addition, the human factor is considered one of the most important factors influencing the formation of soil affected by salt in the Nile Delta. It is noted that the large urban activity in the North Coast region (Alexandria – Matrouh) destroyed living organisms. Abdelaal *et al.* [82] found that the results of the concentrations of total dissolved salts for soil samples collected from 84 soil samples in the eastern Nile Delta showed a large variation in total dissolved solids (T.D.S.), ranging between 3347.2 and 46028.8 mg/l. While on the Cairo-Alexandria desert road, it ranged between 301.76–178848 mg/l. In Siwa Oasis, salinity ranged between 6,425–28 mg/l, while in Fayoum, it ranged between 3,296–177,376 mg/l. There are several reasons for the increased soil salinity in the study area. Urban expansion has led to the loss of fertile land to urban settlements, resulting in a deterioration in soil quality. Groundwater salinity and increased soil salinity are also major concerns, as they lead to salt accumulation, increased electrical conductivity, and changes in soil structure Moursy *et al.* [96], these processes are particularly pronounced in surface soil horizons, leading to increased salinity that weakens soil fertility through processes such as increased sodicity, which reduces water infiltration and hinders root growth. Therefore, these changes pose a significant challenge to agricultural productivity, underscoring the urgent need for effective management practices to monitor soil salinity and soil health, thereby achieving sustainable land use [89].

### 3- Soluble ions

#### A-Soluble cations (Ca^++^, Mg ^++^, Na^+^, and K ^+^).

The calcium content in the soil of the North Coast ranged from a minimum of 3.03 meq/l to a maximum of 3.53 meq/l. This appears to be a low level of calcium, which is partially consistent with the study by Al-sodany *et al.* [97], where calcium content ranged between 0.05 and 43 meq/l. On the Cairo-Alexandria Desert Road, this level ranged between 2.27 and 181.82 meq/l, while in the Siwa Oasis, it ranged between 28.79 and 142.42 meq/l. This is partially consistent with the study by El-Hassanin *et al.* [85], where calcium values ranged between 25 meq/l. In Fayoum, it ranged between 15.15 and 242.42 meq/l. By comparing the calcium content in the soil of each stand, the data presented revealed significant differences between the calcium content of the soil of the North Coast, the soil of the Cairo-Alexandria Desert Road, the Siwa Oasis, and Fayoum. By comparing the calcium content in the soil of each stand, the data presented showed significant differences between the calcium content of the soil of the North Coast, the soil of the Cairo-Alexandria Desert Road, the Siwa Oasis, and Fayoum.

Magnesium content in the soils of the North Coast ranged between 1.3–133.77 meq/l. In Al-sodany *et al.* [97], the study magnesium values ranged between 0.03–24 meq/l. On the Cairo-Alexandria Desert Road, they ranged between 1.52–285.71 meq/l, and in the Siwa Oasis, between 20.35–766.67 meq/l. From the results, we find that the minimum values are consistent with the study by El-Hassanin *et al.* [85], where the magnesium value was 13.3 meq/l. In Fayoum, the value ranged between 10.82–271.65 meq/l. Significant differences were observed between most stands. Comparing the average soil magnesium content for each stand, the same trend was observed with the average soil calcium content. The sodium content in the soil of the North Coast ranged between 0.61–146.09 meq/ l. The sodium value in Al-sodany *et al.* [97], study ranged between 0.04–80.3 meq/l. On the Cairo-Alexandria Desert Road, it ranged between 0.89–3,500 meq/l. In the Siwa Oasis, it ranged between 44.35–3,000 meq/l, and in Fayoum, it ranged between 20.87–4,700 meq/l. Potassium content in the soil of the North Coast ranged between 0–3.54 meq/l. On the Cairo-Alexandria Desert Road, it ranged between 0.15–46.45 meq/l, and in the Siwa Oasis, it ranged between 0.76–126.69 meq/l. In Fayoum, it ranged between 0.55–40.53 (Table 4).

#### B- Soluble anions (CO_3_^=^, HCO_3_^-^, Cl^-^, and SO_4_^=^).

As shown in Table 4, soil bicarbonate levels on the North Coast ranged between 1.18–10.38 meq/l. On the Cairo-Alexandria Desert Road, they ranged between 1.42–6.13 meq/l, and in Siwa, between 2.36–5.66 meq/l. El-Hassanin *et al*. [85], measured bicarbonate levels in the Siwa Oasis at 1.4 meq/l. In Fayoum, they ranged between 2.36–12.74 meq/l. Significant differences exist between stands.

Chloride ions are known to be common in saline soils. Soil chloride levels ranged between 2.54–222.03 meq/l on the North Coast, and between 3813.56–2.54 meq/l on the Cairo-Alexandria Desert Road. In Siwa Oasis, it ranged between 3050.85–59.32 meq/l. This is partially consistent with the study by El-Hassanin *et al.* [85], in which chloride concentrations reached 60 meq/l.In Fayoum, it ranged between 26.27–4372.88 meq/l. Soil sulfate ranged between 1.42–85.3 meq/l on the North Coast. On the Cairo-Alexandria Desert Road, it ranged between 1.04–221.63 meq/l. In Siwa, it ranged between 41.75–572.64 meq/l. This is partially consistent with the study by El-Hassanin *et al.* [85], which was close to the lower limit of the current study’s results, which was 37 meq/l. In Fayoum, it ranged between 19.69–428.92 meq/l.

#### 4-Calcium Carbonate (CaCO_3_%).

Calcium carbonate (CaCO_3_) is valued in soil samples taken from 70 studied stands, ranging between 6.12–80.4% on the North Coast. We found discrepancies in the results when comparing the current study with Al-sodany *et al.* [97], study, where the content ranged between 1.1–17.3%, and with Abdelaal *et al.* [82], study, where the CaCO_3_ value ranged between 1.45–19.31%. On the Cairo-Alexandria Desert Highway, the CaCO_3_ content ranged between 44.5–34.37%. This contrasts with the study by Alnaimy *et al.* [98], where the calcium carbonate content ranged between 23.1 and 14.6%. In Siwa, it ranged between 44.12–45.40%, and in Fayoum, the value ranged between 78.7–89.24%. Alnaimy *et al.* [98] explain this discrepancy by the fact that several factors control the concentration of calcium carbonate in soil, including irrigation water, where CaCO_3_ content decreases with increasing salinity. Furthermore, CaCO_3_ content is linked to organic matter content, increasing with increasing organic matter.

#### 5- Organic Matter.

The data in Table 4 show the organic matter content in soil samples from the 70 stands included in the current study. On the North Coast, the organic matter content ranged between 0.165–1.005%. This is partially consistent with a study by Bedair *et al.* [84], where soil samples had very low organic matter contents, ranging from 0.03–13.6%, and with Al-sodany *et al.* [97], where soil samples had very low organic matter contents, ranging from 0.1–5.3%. On the Cairo-Alexandria Desert Road, organic matter contents ranged from 0.105–1.155%. These results are consistent with a study by Alnaimy *et al.* [98], which was conducted in the newly reclaimed Nubaria land area where a large agricultural development project is being implemented in the western Nile Delta. The aim was to determine the soil capacity and suitability. Three soil profiles were taken for evaluation, and the organic matter value was found to range between 0.07–0.27%. In the Siwa Oasis, it ranged between 0.615–1.53%, and the organic matter content in Siwa ranged between 0.615–1.53%. Therefore, these results are consistent with the results of the study by Elnaggar *et al.* [83], where the organic matter content in Siwa ranged between 0.09–1.3%. In the Fayoum area, the organic matter content ranged between 0.03–1.17%. This is consistent with Abdel-Fattah *et al.* [86], where the percentage ranged between 0.07–1.77%. The results show that organic matter varied slightly across the study area, not exceeding 1.53%.

#### 6 - Sodium adsorption ratio (SAR).

The Specific Absorption Coefficient (SAR) values in soil samples taken from 70 stands varied widely, ranging from 0.36–19.33 in the North Coast soil (Table 4).

These results are slightly like those of Abdelaal *et al.* [82], who recorded values ranging from 13.36–81.47, and SAR values ranged from 0.55–228.92 in the Cairo-Alexandria Desert Road soil. In the Siwa Oasis, values ranged from 8.11–214.94, while in Fayoum, they ranged from 5.79–801.16. A comprehensive review of the data presented in Table 4 regarding the chemical evaluation of soils taken from the 70 studied stands shows that the results of the analysis coefficient values generally show similarity between the North Coast and the Cairo-Alexandria Desert Road, and between the Siwa Oasis and Fayoum. Most of the lowest values were recorded on the North Coast, while most of the highest values were recorded in Siwa and Fayoum. It was noted that the North Coast recorded the lowest average values for measured soil parameters, while the Siwa and Fayoum Oasis recorded the lowest values. Most of the maximum values were recorded for the averages of measured parameters, such as dissolved solids, electrical conductivity, calcium, magnesium, and chlorine.

#### 7- Macronutrients (available N, P and K).

Nitrogen, phosphorus, and potassium are vital for the health and productivity of plants. They contribute to essential processes such as protein synthesis, energy transfer, root development, water regulation, and disease resistance. Proper management of these nutrients in the soil is crucial for sustainable agriculture and optimal crop production.

### A- Soil Nitrogen content (N ^- -^)

The highest soil nitrogen content was recorded (126 mg/kg) in the North Coast samples (S2, S3). The lowest nitrogen content (38.75 mg/kg) was recorded in the North Coast samples (S23, S26), with an average of 79.452 mg/kg (Table 4). The nitrogen content in the soil of the North Coast ranged between 38.75 and 126 mg/kg, and on the Cairo-Alexandria Desert Road, the content ranged between 51.67 and 103 mg/kg. In Siwa, it ranged between 51.67 and 103 mg/kg, while in Fayoum, it ranged between 64.58 and 90.41 mg/kg. In the study by Abdel-Fattah *et al.* [86], the nitrogen content was low, ranging between 1.33 and 61.55 mg/kg.

#### B- Soil phosphorus content (P ^--^).

In general, the highest soil phosphorus content was (29.03 mg/kg) in the Siwa Oasis sample (S55). The lowest phosphorus content (0.04 mg/kg) was recorded in the North Coast sample (S6) (averaging 4.348 mg/kg). Specifically, phosphorus content on the North Coast ranged between 0.04–22.3 mg/kg, while on the Cairo-Alexandria coastal road, it ranged between 0.07–8.38 mg/kg. In the Siwa Oasis, it ranged between 0.07–29.03 mg/kg, and in Fayoum, it ranged between 1.86–20.91 mg/kg (Table 4). This is partially consistent with the study by Abdel-Fattah *et al.* [86], where phosphorus content ranged between 2.33–19.84 mg/kg.

#### C- Soil Potassium content (K^- -^).

The highest soil potassium content was recorded (1811.76 mg/kg) in Siwa Oasis, sample (S61), while the lowest potassium content (6.41 mg/kg) was recorded in the North Coast, sample (S1). The potassium content in the soil of the North Coast ranged between 6.41–395.31 mg/kg, and on the Cairo-Alexandria Desert Road, it ranged between 32.77–1482.18 mg/kg. In the Siwa Oasis, it ranged between 105.28–1811.76 mg/kg, and in Fayoum, it ranged between 181.08 mg/kg (Table 4). In Fayoum, the potassium content is consistent with the study of Abdel-Fattah *et al.* [86], where it ranged between 32.76–733.77 mg/kg.

### Data treatment and classification

#### TWINSPAN.

**Fig 7:**
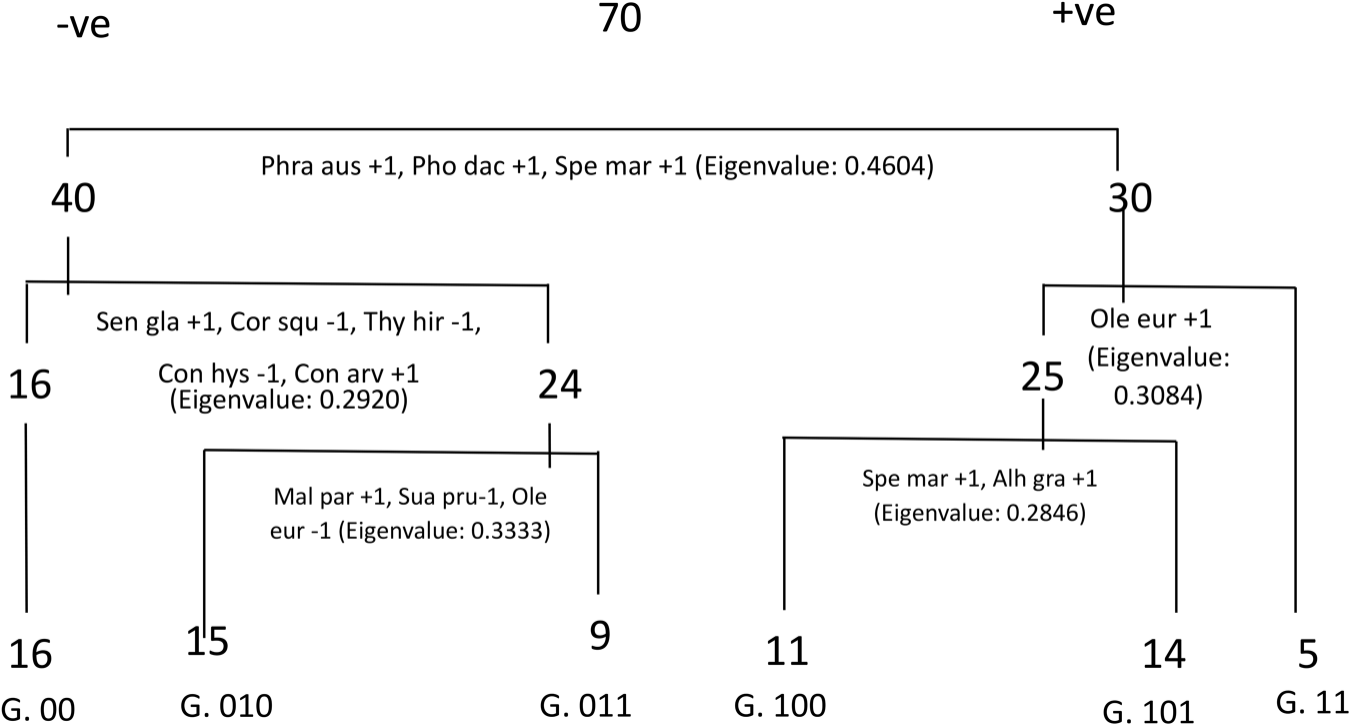
TWINSPAN classification for the vegetation of the selected 70 stands. 00, 010, 11, 011, 100, and 101 were the six separate vegetation clusters.

Two–way indicator species analysis, a divisive cluster analysis method, was used to identify ecological groups depending on indicator species that characterize each group. Depending on species distribution, species groups were also identified for each matrix. Based on the TWINSPAN outcome of the 70 stands of agricultural lands affected by salinity in Egypt during the spring season, using pseudo-species cut levels 0, 1 (present or absent), TWINSPAN divided the stands into six vegetation groups. Each cluster has one or more indicator species that characterize that particular habitat according to the most distinctive species and the most abundant characteristic species that reached the highest IVI values (Fig 7).

For the first level of classification, *Phragmites australis*, *Phoenix dactylifera, Spegularia marina* were the indicator species. were the indicator species for the positive group (30 stands). The 70 stands were separated into two groups (0 and 1). Group 0 comprises 40 stands, while group 1 comprises the rest 30 stands. For the second level of classification and based on *Senecio glaucus*, *Coronopus squamatus*, *Thymelaea hirsuta*, *Convolvulus hystrix*, *Convolvulus arvensis* the 40 stands in group 0 had been further classified into two groups (00 and 01). Group 00 included 16 stands, while Group 01 included 24 stands. *Senecio glaucus, Convolvulus arvensis* was the indicator species for group 01 (24 stands) while *Coronopus squamatus*, *Thymelaea hirsuta*, *Convolvulus hystrix* were the indicator species for group 00 (16 stands). the 30 stands in group 1 had been further classified into two groups (10 and 11). Group 10 included 25 stands, while group 11 included 5 stands. *Olea europaea* was the indicator species for group 11 (5 stands). For the third level of classification, and based on *Malva parviflora*, for the positive group (010) (15 stands) and *Olea europaea* for the negative group (011) (9 stands).

Group 010: 15 stands (2, 3, 9, 10, 14, 23, 29, 40, 41, 42, 43, 45, 49, 50 and 52), Group 011:9 stands, (1, 4, 8, 11, 13, 26, 44, 47 and 48), Group 00: 16 stands (5,6,7,12,15,16,17,18,19,20, 21, 22, 24, 25,38 and 46), Group 11: 5 stands (51, 64, 65, 66 and 70), and Group 101: 14 stands (stand 39,53–63, 67and 69).

### Detrended Correspondence Analysis

**Fig 8:**
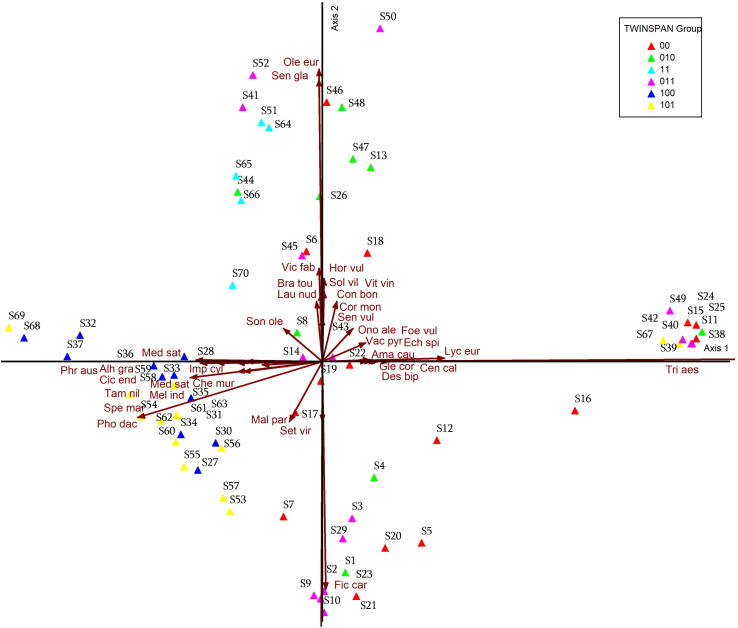
DCA ordination diagram for the TWINSPAN groups, with different plant indicators.

**Fig 9:**
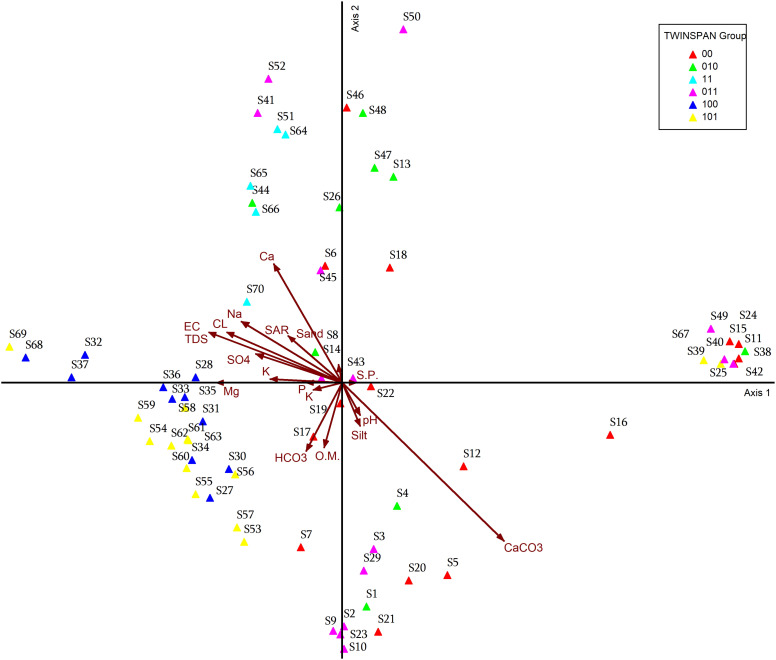
DCA ordination diagram for the TWINSPAN groups, with different plant indicators.

**Fig 10:**
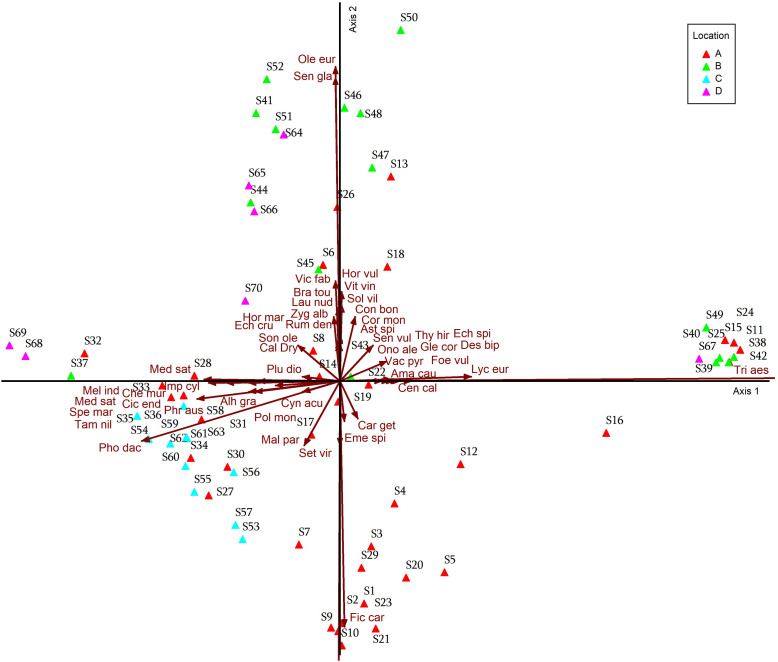
DCA ordination diagram for the location groups of the 70 stands. On axes 1 and 2 in relation to different plant indicators.

**Fig 11:**
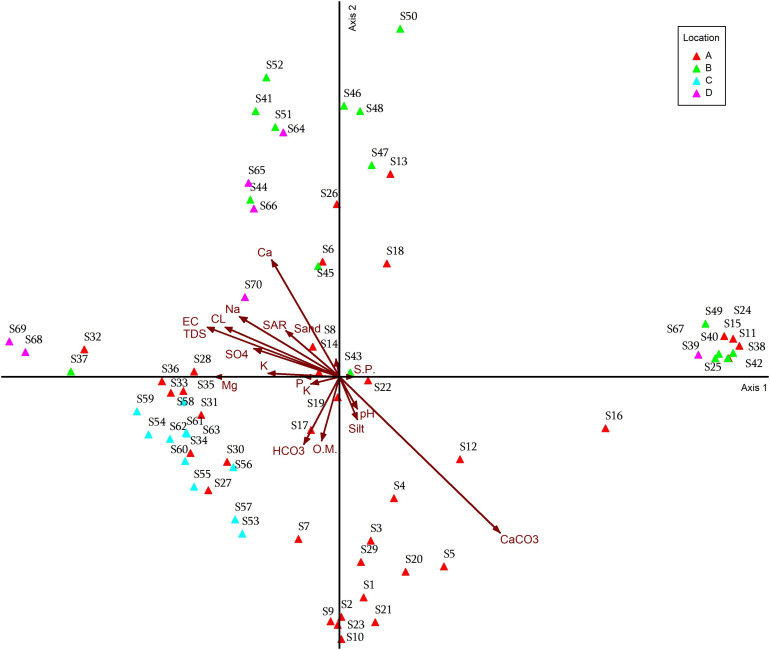
DCA ordination diagram for the vegetation of the 70 stands with location.

DCA was applied to data of agricultural lands affected by salinity in Egypt, and results were plotted into DCA graphs in Figs 8–11. When plotted on the first two DCA ordination axes, stands tend to cluster into the six vegetation groups resulting from TWINSPAN, which were described before. Ordination graphs showed clearly which stands were transitional in their composition within TWINSPAN groups. The most influential plant indicators associated with the group of stands located within the TWINSPAN groups as follows: the most influence indicator plants in the group of stands belonging to group 101 (S53-S63), group 100 (S27, S30, S31, S33, S34, S35, S36), group 00 (S7, and S17), group 011(S9) were: *Alhagi graecorum, Chenopodium murale, Cichorium endivia, Imperata cylindrica, Malva parviflora, Melilotus indicus, Phoenix dactylifera, Phragmites australis, Setaria viridis, Spergularia marina, Tamarix nilotica* (Fig 8). While the following plant indices *Brassica tournefortii, Launaea nudicaulis, Sonchus oleraceus, Vicia faba, Medicago sativa, Olea europaea, Senecio glaucus* are most closely related to group 100 (S28, S32, S36, S37, S68), group 11 (S51, S64, S65, S66, S70), group 010 (S8, S44), group 011 (S14, S41, S45, 52), group 00 (S6) and group 101 (S69). It is clear that the plant indicators most closely related to group 00 (S5, S12, S16, S19, S20, S21), group 010 (S2, S4) and group 011 (S3, S10, S29) are: *Glebionis coronaria, Desmostachya bipinnata, Ficus carica,* and *Centaurea clacitrapa.* On the other hand, the most influential indicator plants were in the group of stands belonging to the following twin groups: group 00 (S24, S25, S46, S18, S15, S11), group 010 (S38, S48, S47, S13), group 011 (S50, S49, S42), and group 101 (S39, S (67 *Hordeum vulgare, Solanum villosum, Conyza bonariensis, Cornulaca monacantha, Onopordum alexandrinum, Lycium europaeum, Foeniculum vulgare, Senecio vulgaris, Echinops spinosus, Vaccaria pyramidata, Vitis vinifera,* and *Amaranthus caudatus.*

While Group 00 (S6), Group 011 (S41, S45, S52), Group 010 (S14, S26, S44), Group 11 (S51, S64, S65, S66, S70), Group 100 (S68, S37, S32, S36, S28), and Group 101 (S69). These groups are more closely related to the environmental factors represented by Electrical conductivity, total dissolved salts, sulfate, adsorption rate of sodium, chloride, calcium, sodium, potassium, and sand Group 100 (S27, S30, S31, S34, S35, S33, S36, S59, S58) and group 101 (S53-S63), group 00 (S7, S17, S19), group 011 (S9) these groups are most closely related to environmental factors: bicarbonate, organic matter, magnesium, potassium, and phosphorus. As for the 00 group (S5, S12, S16, S20, S21, S22), the 010 group (S1, S4), and the 011 group (S2, S3, S9, S10, S29), the environmental factors most closely related to them are calcium carbonate, pH, and silt. On the other hand, find the 00 group (S11, S15, S18, S42, S46), the 011 group (S50, S43, S49, S25, S42), and the 010 group (S48, S47, S13, S38). These groups are more closely related to saturation percentage (Fig 9).

The most closely associated plant indices for each group of stands. The results showed that some stands on the North Coast (S7, S27, S30, S31, S33, S34, S35, S36), and all studied stands in Siwa (S53-S63), had the most closely associated plant indices: *Alhagi graecorum, Chenopodium Murale, Cichorium endivia, Cynanchum acutum, Imperata cylindrica, Malva parviflora, Medicago sativa, Melilotus indicus, Phoenix dactylifera, Phragmites australis* (Fig 10). On the other hand, the plant indices most closely associated with some stands on the North Coast (S6, S8, S14, S26, S28, and S32), some stands on the Cairo-Alexandria Desert Road (S37, S41, S44, S45, S51, and S52), and most stands in Fayoum (S64, S65, S66, S68, S69, and S70) are *Brassica tournefortii, Calotropis procera, Echinochloa crus-galli, Hordeum marinum, Launaea nudicaulis, Medicago sativa, Pluchea dioscoridis, Rumex dentatus, Sonchus oleraceus, Zygophyllum album, and Vicia faba.* While *Hordeum vulgare, Vitis vinifera, Triticum aestivum, Astragalus spinosus, Conyza bonariensis, Cornulaca monacantha, Desmostachya bipinnata, Echinops spinosus, Foeniculum vulgare, Glebionis coronaria, Lycium europaeum, Onopordum alexandrinum, Senecio vulgaris, Solanum villosum, Thymelaea hirsuta, Vaccaria pyramata* were the most influential indicator plants in some stands on the North Coast (S11, S13, S18, S24, S25), most stands on the Cairo-Alexandria Desert Road (S38-S40, S42, S43, S46-S50), and one stand in Fayoum (S67). *Ficus carica, Carduus getulus, Centaurea calcitrapa, Emex spinosa* were the most influential indicator plants in the North Coast at stands (S1-S5, S9, S10, S12, S20, S21, S23, S29).

The relationship between the study stands and environmental factors (soil factors), i.e., the soil properties most closely associated with the stands in each study area. Regarding the environmental factors, find that some stands on the North Coast (S6, S8, S14, S26, S28, and S32), some stands on the Cairo-Alexandria Desert Road (S37, S41, S44, S45, S51, and S52), and some stands in Fayoum (S64, S65, S66, S68, S69, and S70) were most closely associated with environmental factors (soil) with respect to the following properties: electrical conductivity, total dissolved salts, sodium absorption rate, sand, positive ions (sodium, calcium, potassium), and negative ions (sulfates, chlorides). As for the Siwa stands (S53-S63), and some North Coast stands (S7, S27, S9, S30, S31, S33, S34, S35, S36), they were most closely related to the following soil properties: phosphorus, potassium, magnesium, bicarbonate, and organic matter. Some North Coast stands (S1-S5, S9, S10, S12, S20, S21, S23, S29) were uniquely related to the following environmental factors: pH, calcium carbonate, and silt. The remaining North Coast stands (S11, S13, S18, S24, S25), the remaining Cairo-Alexandria Desert Road stands (S38-S40, S42, S43, S46-S50), and one Fayoum stand (S67) were most strongly related to a single soil factor (saturation percentage) (Fig 11).

Detrended Correspondence Analysis (DCA) is a commonly employed ordination method in ecology. It serves to identify distinct plant communities by measuring changes in species composition associated with underlying environmental factors. Many researchers have used this analysis (DCA) and principal component analysis (PCA) to determine the most influential environmental factors in the distribution of plant communities [99–103]. The results of the current study indicate that the environmental factors most closely related to some clusters on the North Coast, some clusters on the Cairo-Alexandria Desert Road, and some clusters in Fayoum are electrical conductivity, total dissolved solids, sodium absorption rate, sand, positive ions (Na, Ca, K), and negative ions (Sulfate and chloride). This is partially consistent with several studies, including the study conducted by [104], on vegetation cover and soil morphology in 10 stands in Wadi El Rayan Protected Area, Western Desert, Egypt. Using multivariate methods, researchers concluded that soil properties, such as pH, electrical conductivity, calcium carbonate, organic matter, and cations, played an important role in vegetation species pattern and vegetation organization. El-Sayed *et al.* [105] studied plant-soil relationships in southwest Sinai and concluded that soil properties, such as gravel content, organic carbon, pH, and sand percentages, corresponded to the different plant groups classified according to the Twinspan system. Salama *et al.* [106] also studied plant-soil relationships within a wadi system in the Eastern Central Desert of Egypt, assessing the distinct role of soil moisture content, salinity, and fertility status in regulating plant communities. Among the Siwa stands and some North Coast stands, the following soil properties were closely related: phosphorus, potassium, magnesium, bicarbonate, and organic matter.

These results are consistent with several studies, including one by El-Zeiny *et al.* [36], which used remote sensing and GIS modeling to map terrestrial plant habitats in the Mediterranean coastal region of Egypt, the North Coast. In this study, natural vegetation trends were identified as strongly related to soil phosphorus, potassium, magnesium, and organic matter content, which support plant growth in these semi-arid coastal areas. The addition of the NDVI index and soil surface temperature to the analysis improved habitat suitability models for some feral plants and emphasized the importance of these soil properties in maintaining vegetation in the region. The study by Abd El-Ghani *et al.* [78] conducted a detailed analysis of floristic diversity and vegetation cover in the Siwa Oasis, demonstrating that soil salinity and moisture gradients are the main factors controlling vegetation patterns. Their study used multivariate analyses (DCCA, TWINSPAN) and found that organic matter and fine soil fractions were closely related to vegetation groups.

Studies conducted by Salem & Jia [107] confirmed problems with the sustainability of vegetation and soil cover in the Siwa Oasis, reporting increased soil salinity due to over-irrigation and rising water levels, which negatively impact vegetation cover and species distribution. They highlighted the critical importance of soil chemical properties for ecosystem health. Some northern coastal plants were independently associated with the following environmental factors: pH, calcium carbonate, and silt. Other North Coast plant groups, other Cairo-Alexandria Desert Road plant groups, and the Fayoum plant group were most closely related to a single soil factor (saturation percentage). Salama *et al.* [108] studied the Egyptian deserts (Eastern and Western Deserts). Six distinct plant groups, each with a distinct vegetation composition, were identified after a TWINSPAN plant taxonomy study. Sand, clay, water content, organic matter, sodium, potassium, calcium, and magnesium were strongly associated with the first four frequency axes, accounting for 76.1% of the species-environment relationships among the studied groups.

El-Sayed *et al.* [105] studied plant-soil relationships in southwest Sinai and found that soil properties, such as gravel content, organic carbon, pH, and sand fraction, are associated with different plant communities identified by the TWINSPAN program. Abbas *et al.* [109] analyzed soil and plant composition at 10 stands in Wadi El Rayan, Fayoum. Using TWINSPAN, they classified the communities into four plant groups, each associated with specific dominant species and habitat types (e.g., wetlands, sabkhas, and sand dunes). The study found that differences in soil texture (mainly sandy and loamy), saturation ratio, and other physical properties were closely related to the distribution of plant communities. Salama *et al.* [52] analyzed plant-soil interactions in a wadi system in the central Eastern Desert of Egypt and tested the distinct role of soil moisture content, salinity, and fertility status in controlling plant communities.

## Supporting information

S1 File Supplementary File. (DOCX)

S2 File Vegetation and Soil Analyses. (XLS)

S3 File Vegetation and Soil Analyses (compressed file). (ZIP)
