# Supplementary material for: Evaluation of plant diversity and soil-vegetation relationships in some salinity-affected cultivated areas in Egypt
Source: PLoS One. 2026 Apr 24;21(4):e0346662. doi: 10.1371/journal.pone.0346662 (PMC13108757; doi:10.1371/journal.pone.0346662)
Supplement: S1 File — (DOCX) [file pone.0346662.s001.docx]

**Materials and Methods**

No permits were required from any authority because this study focused on agricultural lands affected by salinity in predetermined locations. All the agricultural lands visited were privately owned, and the owners were interviewed during the field visits and given verbal consent for the field study and the collection of plant and soil samples.

**Study area**

The global positioning system (GPS) coordinates of the 70 studied stands are provided in Table S1.

Table S1. Global Positioning System (GPS) coordinates of the 70 stands selected in the different study areas

| Stands | Latitude (N) | Longitude (E) | Stand Name | Location |
| --- | --- | --- | --- | --- |
| 1 | 30.98408 | 29.58241 | Borg El Arab Airport Road | North Coast |
| 2 | 30.95738 | 29.53656 | Borg El Arab |  |
| 3 | 30.95738 | 29.53656 | Borg El Arab |  |
| 4 | 31.01598 | 29.6352 | Borg El Arab |  |
| 5 | 31.0367 | 28.45703 | El-Hamam |  |
| 6 | 31.03929 | 28.44624 | El-Hamam |  |
| 7 | 31.01298 | 28.58871 | El-Alamein |  |
| 8 | 30.88646 | 28.85794 | El-Alamein |  |
| 9 | 30.82771 | 29.20371 | Sidi Abd El-Rahman |  |
| 10 | 30.95738 | 29.53656 | Sidi Abd El-Rahman |  |
| 11 | 30.83401 | 28.96216 | El-Dabaa, Matrouh |  |
| 12 | 31.03929 | 28.44624 | El-Dabaa, Matrouh |  |
| 13 | 31.03887 | 28.44824 | El-Dabaa, Matrouh |  |
| 14 | 31.03929 | 28.44624 | El-Dabaa, Matrouh |  |
| 15 | 31.03929 | 28.44624 | El-Dabaa, Matrouh |  |
| 16 | 31.25666 | 27.29695 | Mersa Matrouh |  |
| 17 | 31.269901 | 27.09334 | Mersa Matrouh |  |
| 18 | 31.279575 | 27.05187 | Mersa Matrouh |  |
| 19 | 31.293182 | 27.01153 | Mersa Matrouh |  |
| 20 | 31.302784 | 26.9697 | Mersa Matrouh |  |
| 21 | 31.400008 | 26.60723 | Mersa Matrouh |  |
| 22 | 31.278535 | 27.30388 | Mersa Matrouh |  |
| 23 | 31.200378 | 27.44071 | Mersa Matrouh |  |
| 24 | 31.185364 | 27.54512 | Mersa Matrouh |  |
| 25 | 31.141922 | 27.67624 | Mersa Matrouh |  |
| 26 | 31.12923 | 27.73085 | Mersa Matrouh |  |
| 27 | 31.301338 | 30.26689 | Al Miaddiyyah |  |
| 28 | 31.291458 | 30.24018 | Rashid |  |
| 29 | 31.404635 | 30.35794 | Al Gazirah Al Khadraa, Kafr El Sheikh |  |
| 30 | 31.451801 | 30.44 | Al-Hdddiadi, Kafr El Sheikh |  |
| 31 | 31.446822 | 30.59003 | Sidi Salem, Kafr El Sheikh |  |
| 32 | 31.490746 | 30.73525 | Ezbet Halawa, Kafr El Sheikh |  |
| 33 | 31.527919 | 30.82211 | Al Albaad, Kafr El Sheikh |  |
| 34 | 31.559519 | 30.91584 | Al Bananin, Kafr El Sheikh |  |
| 35 | 31.559519 | 31.18775 | Baltim, Kafr El Sheikh |  |
| 36 | 31.478449 | 31.40611 | Belqas, Dakahlia |  |
| 37 | 30.2356 | 30.6742 | Cairo-Alexandria Desert Road | Cairo-Alexandria Desert Road |
| 38 | 30.408672 | 30.37002 | Cairo-Alexandria Desert Road |  |
| 39 | 30.520236 | 30.24076 | Cairo-Alexandria Desert Road |  |
| 40 | 29.90907 | 30.83849 | Al Mahdeyah, Beheira |  |
| 41 | 30.611286 | 30.13433 | Cairo-Alexandria Desert Road |  |
| 42 | 30.17185 | 30.91176 | Abu Al Matamir, Beheira |  |
| 43 | 30.10656 | 30.64476 | Abu Al Matamir, Beheira |  |
| 44 | 30.16478 | 30.48619 | Wadi El Natrun-El Alamein Road |  |
| 45 | 30.21131 | 30.50217 | Wadi El Natrun-El Alamein Road |  |
| 46 | 29.89388 | 30.58631 | Wadi El Natrun-El Alamein Road |  |
| 47 | 30.739361 | 30.022586 | Wadi El Natrun |  |
| 48 | 30.4925 | 30.191192 | Wadi El Natrun |  |
| 49 | 30.270111 | 30.537389 | Wadi El Natrun-El Alamein Road |  |
| 50 | 30.600833 | 30.207969 | El-Hamam-Wadi El Natrun Road |  |
| 51 | 30.507082 | 30.3322 | Cairo-Alexandria Desert Road |  |
| 52 | 30.329806 | 30.44063 | Cairo-Alexandria Desert Road |  |
| 53 | 29.246 | 25.574 | Abu Shabwna | Siwa |
| 54 | 29.254168 | 25.59972 | Bastona |  |
| 55 | 29.250834 | 25.70611 | Abu-Elief |  |
| 56 | 29.227 | 25.72 | El-Maamal |  |
| 57 | 29.210687 | 25.5435 | Aghurmi |  |
| 58 | 29.187546 | 25.74174 | Al-Awsat |  |
| 59 | 29.238 | 25.439 | Ain Cleopatra |  |
| 60 | 29.244 | 25.461 | Nejden |  |
| 61 | 29.233145 | 25.48094 | Wazidi |  |
| 62 | 29.233511 | 25.66306 | El-Tabu |  |
| 63 | 29.22097 | 25.51478 | Abu-Bakr |  |
| 64 | 29.54592 | 30.82144 | Madinah Farm | Fayoum |
| 65 | 29.516197 | 30.82934 | Ezbet Farag El-Nuweishi |  |
| 66 | 29.493563 | 30.82779 | Ezbet Saleh Shamata |  |
| 67 | 29.479741 | 30.82384 | Ezbet Ahmed Youssef |  |
| 68 | 29.473838 | 30.81217 | Ezbet Atiya Al-Awkali |  |
| 69 | 29.470401 | 30.80196 | Monshaat El-Sadat |  |
| 70 | 29.466067 | 30.74522 | Ezbet Abd El Qawy Shaaban |  |

**Geology and Geomorphology**

Regarding the Northwest Coast, the Nile Delta, carbonate ridges form a distinctive feature of Egypt's northwestern coast, a geomorphic characteristic not found elsewhere in the country. These ridges, together with the intervening low-lying areas, form a localized landscape known as the Ridge-Depression System. This system runs roughly parallel to the Mediterranean coastline from Alexandria in the east to the city of Salloum in the west. It consists of a series of low-relief ridges, varying in number locally. Ridge elevation ranges from approximately 10 to 25 meters above mean sea level for the first coastal ridge, up to 110 meters for the eighth inland ridge. Ridge width varies between approximately 100 and 10,000 meters. Some ridges are now submerged beneath the Arab Gulf and Maryut Lake or buried by Nile sediments northwest of the Nile Delta [S1]. The coastal fringes of the delta, extending approximately 10 kilometres inland and not exceeding 3.0 meters above mean sea level, feature distinct landforms shaped by the interaction between land and sea. This unique environment is the result of the combined influence of multiple factors, both historical and contemporary. These include sea-level fluctuations, climatic variations impacting Nile discharge and sediment supply, erosion by eastward-directed coastal and offshore currents, human activities, prevailing wind regimes, and neotectonic movements. The interplay of these diverse forces has produced a suite of coastal geomorphic features, creating an environmental zone distinct from the broader deltaic plain. Characteristic landforms within this zone are the coastal plain, lagoons, dunes, and the Barari (wasteland) flats [SI].

The Cairo-Alexandria desert road, between 135 km and 163 km north of Cairo, crosses an area of aeolian sands characterized by undulating topography. Dunes of varying heights are present, decreasing in size northward and eventually diminishing to a shallow layer of sand overlying the compact soils of the northern sector. Soils are very deep and coarse-textured, becoming progressively finer-textured toward the north. The coarse wind-deposited sand is mixed with terrestrial snail shell fragments, which occur in significantly greater abundance in the northern part. The landscape is yellowish-brown, darkening progressively from north to south. Adjacent to the section from 163 km to 170 km north of Cairo, the terrain becomes nearly level, covered by a very shallow and variably thick layer of windblown sand. Hardened calcareous concretions overlie the surface. Most perennial vegetation in this zone exhibits a mound-forming growth habit. From approximately 170 km northward to Alexandria, the area forms part of the coastal belt. A prominent ridge is located here, upon which the town of Amiriya is situated (approximately 34 km southwest of Alexandria). The northern foot of this ridge borders Lake Mariut, which is itself bounded on its north side by further ridges that extend to the coastal plain [S2].

Siwa Oasis is the farthest oasis depression from the Nile Valley to the west and is located approximately 300 km south of the Mediterranean coast (from 29°10' to 29°16' N Latitude and 25°27' to 25°35' E Longitude). The depression is approximately 50 km in length, varying from 2 to 20 km in width, and encompasses about 1000 km^2^. Siwa Oasis extends between 0 and 18 meters below sea level [S3] and displays numerous landforms, including salt lakes, salt marshes (Sabkhas), as well as cultivated lands and orchards [S4]. The region is hyper-arid, receiving 10 mm or less than average annual precipitation, and evaporation rates are in the vicinity of 3000 mm per annum [S5]. One of the features that characterizes Siwa Oasis is the salt lakes. In the west lie Birket Al-Maraqi, 9 km^2^, and Birket Siwa at 32 km^2^ (the largest lake in the depression). In the east lie Birket Zaytun, 16 km^2^, and Birket Azmuri [S6].

Of the major depressions in Egypt's Western Desert, the Fayum Depression is the closest to the Nile Valley. They are separated only by a divide 1.5 to 10 kilometres wide, which allows for the passage of Nile water into the depression. This proximity distinguishes Fayum from the other large depressions in the region. The depression is excavated within a broad dome composed of Eocene and Oligocene rock strata, which are overlain by volcanic materials. Miocene sediments are not present, as the Fayum dome remained above the level of the Miocene Sea. All Pliocene deposits here are continental and fluvial in origin. The Pleistocene deposits are entirely derived from the Nile, which entered the depression via the Hawara Channel during the Early Palaeolithic period, leading to the formation of several lakes. Human intervention during the Ptolemaic Period regulated the water entering the depression according to agricultural needs, causing the lake to shrink to its current level of 45 meters below sea level. Following this, new geomorphic features appeared on the depression floor. Alongside pre-existing forms such as ancient lake beaches, the inverted wadis on the Nile-Fayum divide, and the Hawara Channel (which originated in pre-Ptolemaic times), new features were created. These include Lake Qarun, the Fayum Delta built from Nile silt, and canyon-like wadis [S1].

**Climate**

Based on 30-year climatic data, the regions exhibit distinct patterns: in the North Coast, mean maximum temperatures range from 18°C (January) to 23°C (August), while mean minimums range from 10°C (January) to 23°C (July-August); along the Cairo-Alexandria Desert Road, mean maximums vary from 19°C (January) to 34°C (August) and mean minimums from 9°C (January) to 22°C (July-August); in Siwa, mean maximums extend from 18°C (January) to 37°C (July-August) with mean minimums from 7°C (January) to 24°C (August); and in Fayoum, mean maximums range from 20°C (January) to 37°C (July-August) with mean minimums from 9°C (January) to 24°C (July-August). Rainfall is generally low, with the North Coast and Desert Road receiving up to 8 mm in January, while Siwa and Fayoum receive a maximum of 2 mm (in February and March, respectively), and all locations experience multiple consecutive months with 0 mm precipitation. The prevailing wind direction is north-westerly for the North Coast and Desert Road, north-easterly for Siwa, and northerly for Fayoum (Fig. S1-S8) (www.meteoblue.com).


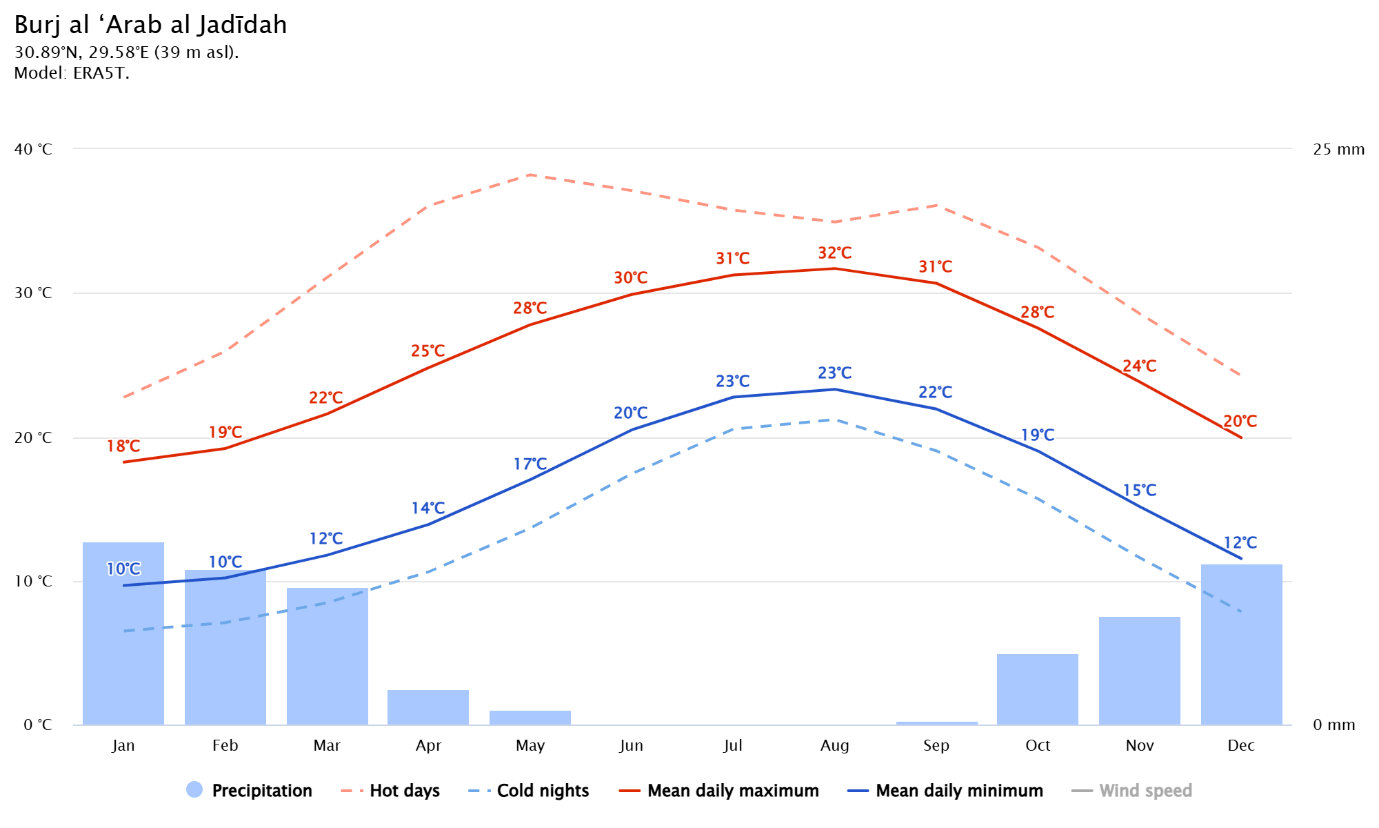


Fig. S1. Klima diagram showing average maximum, minimum temperature, hot days, cold nights, and rainfall in the North Coast.


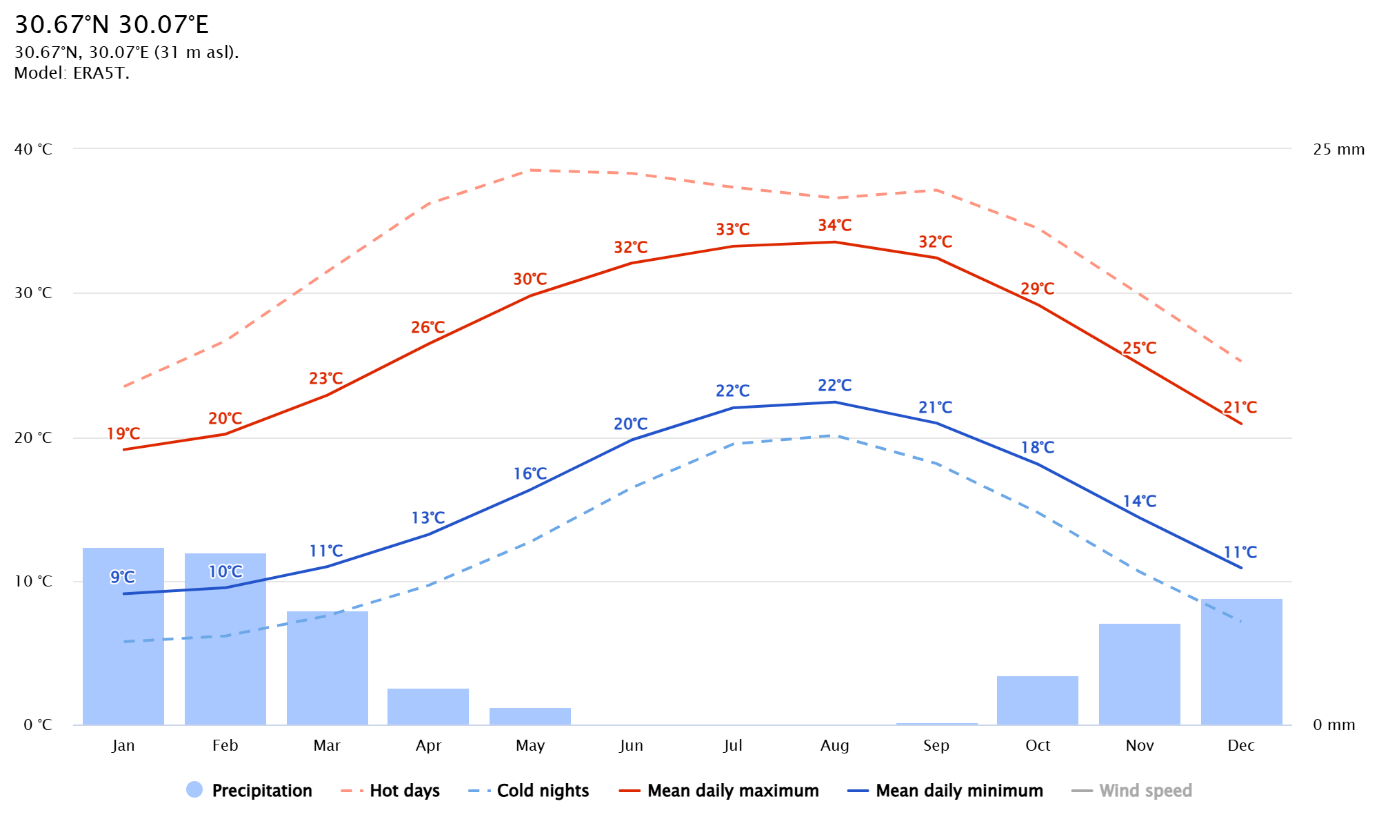


Fig. S2. Klima diagram showing average maximum, minimum temperature, hot days, cold nights, and rainfall in Cairo-Alexandria desert road.


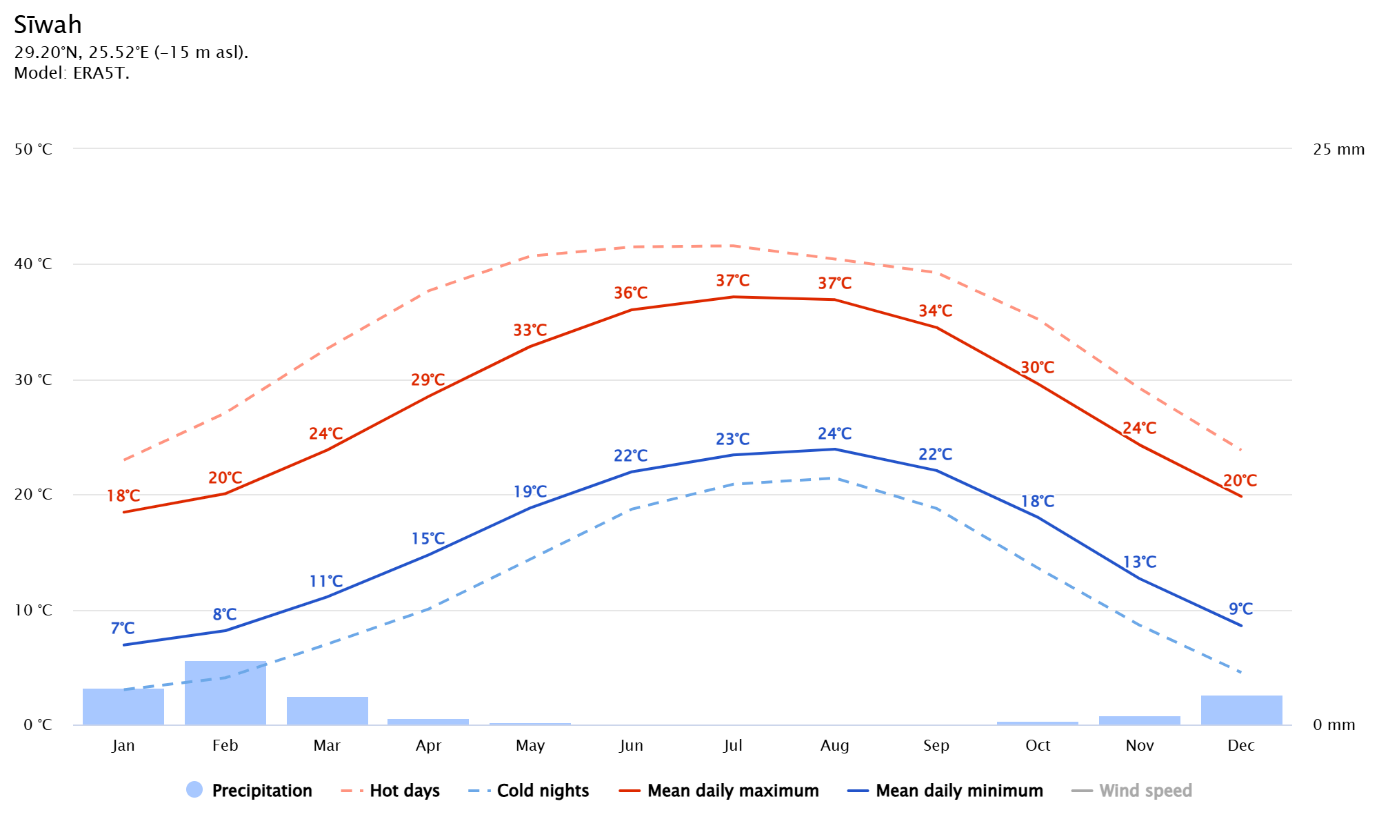


Fig. S3. Klima diagram showing average maximum, minimum temperature, hot days, cold nights, and rainfall in Siwa.


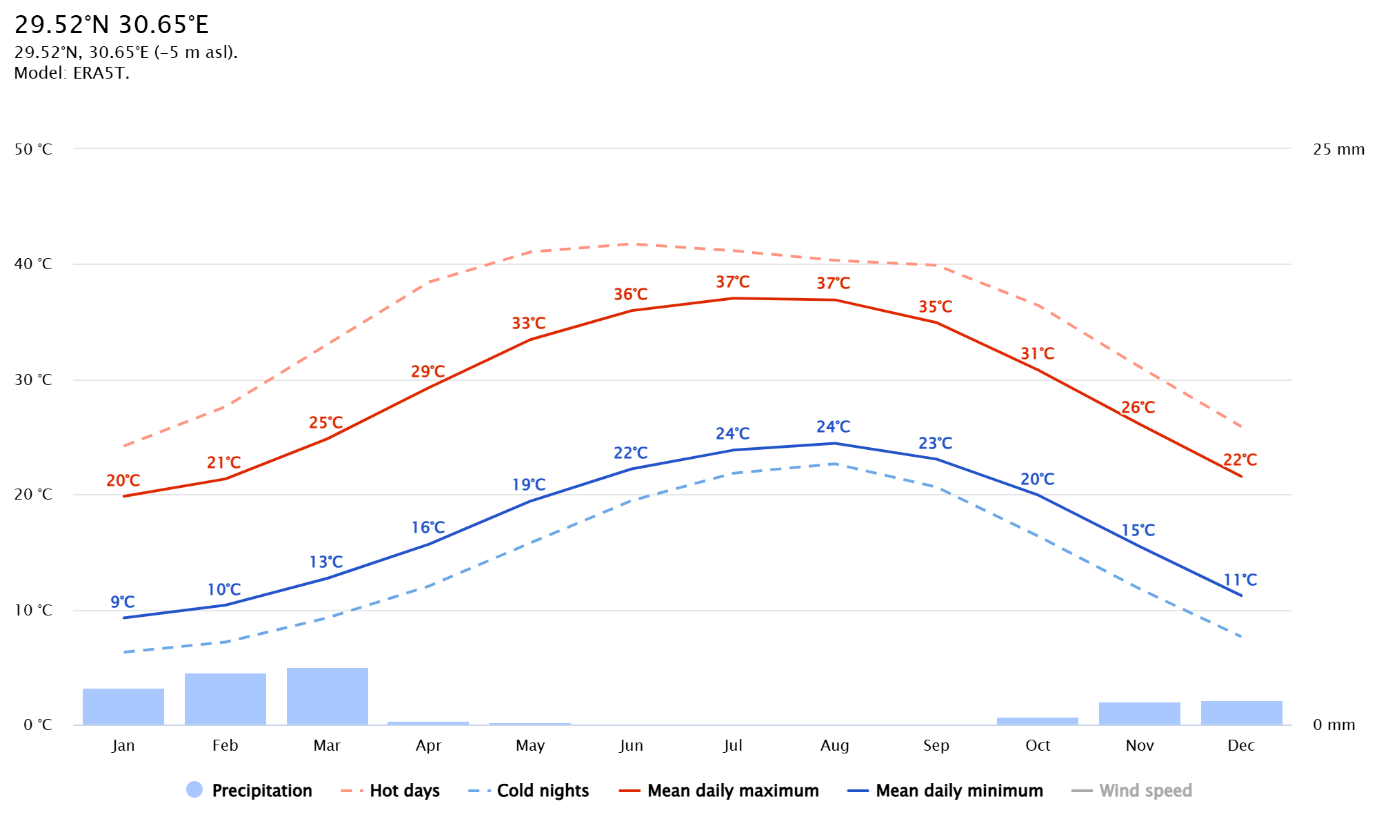


Fig. S4. Klima diagram showing average maximum, minimum temperature, hot days, cold nights, and rainfall in Fayoum.


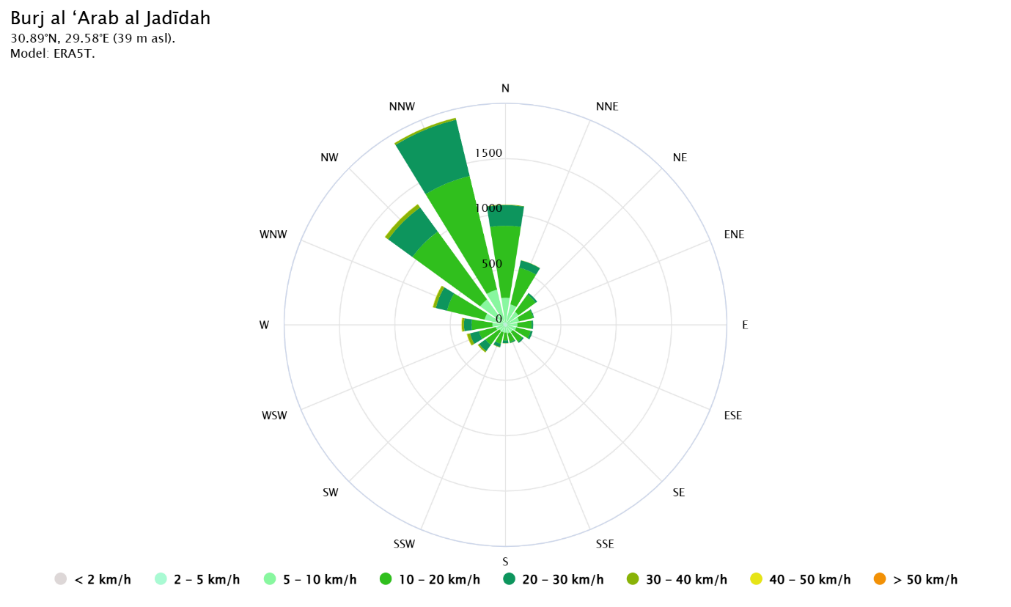


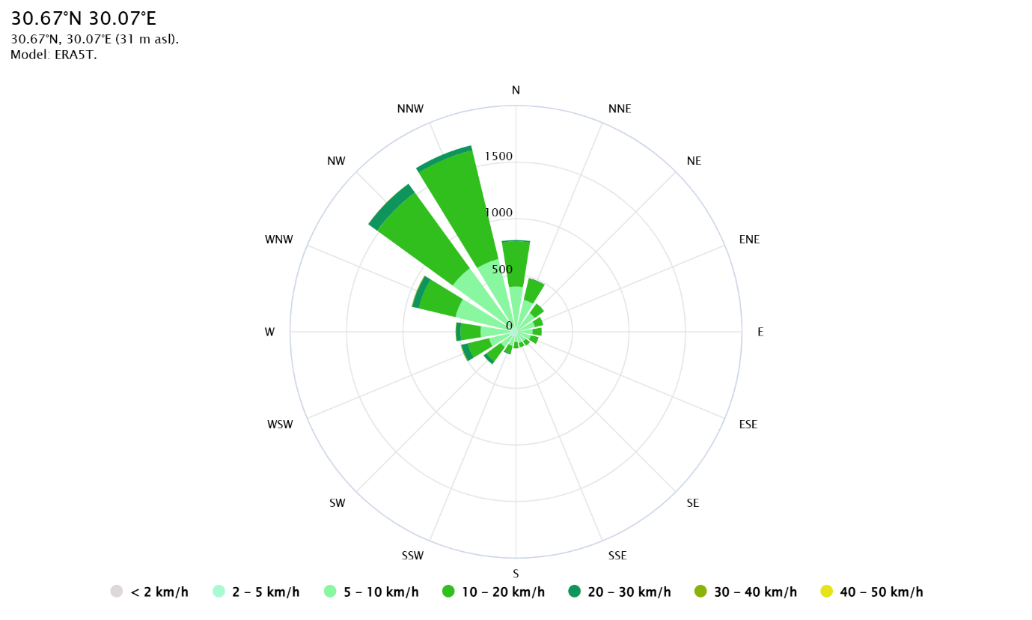
Fig. S5. Wind rose showing the wind direction of the North Coast.

Fig. S6. Wind rose showing the wind direction of the Cairo-Alexandria desert road.


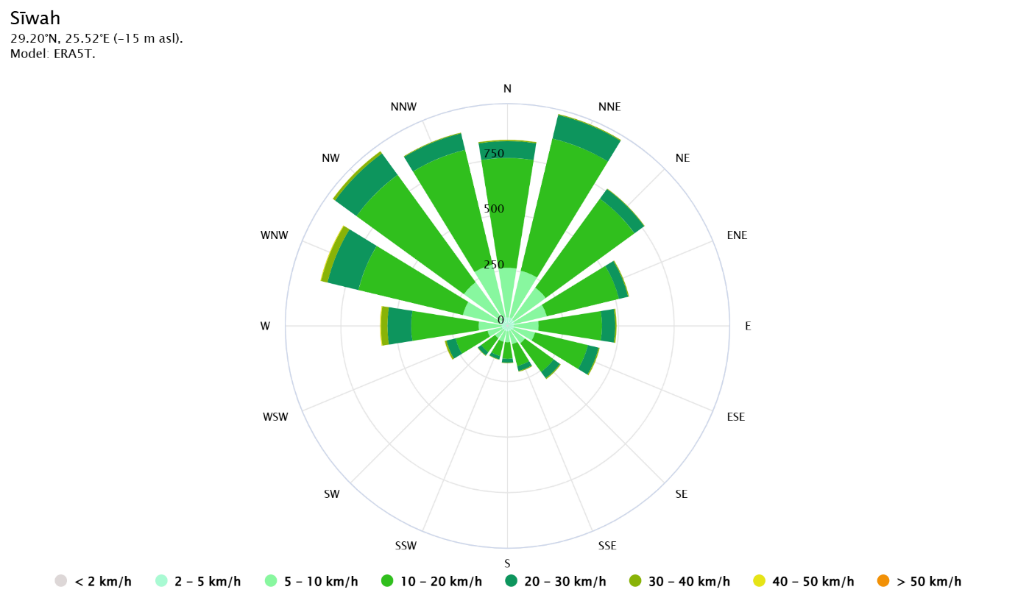


Fig. S7. Wind rose showing the wind direction of Siwa.


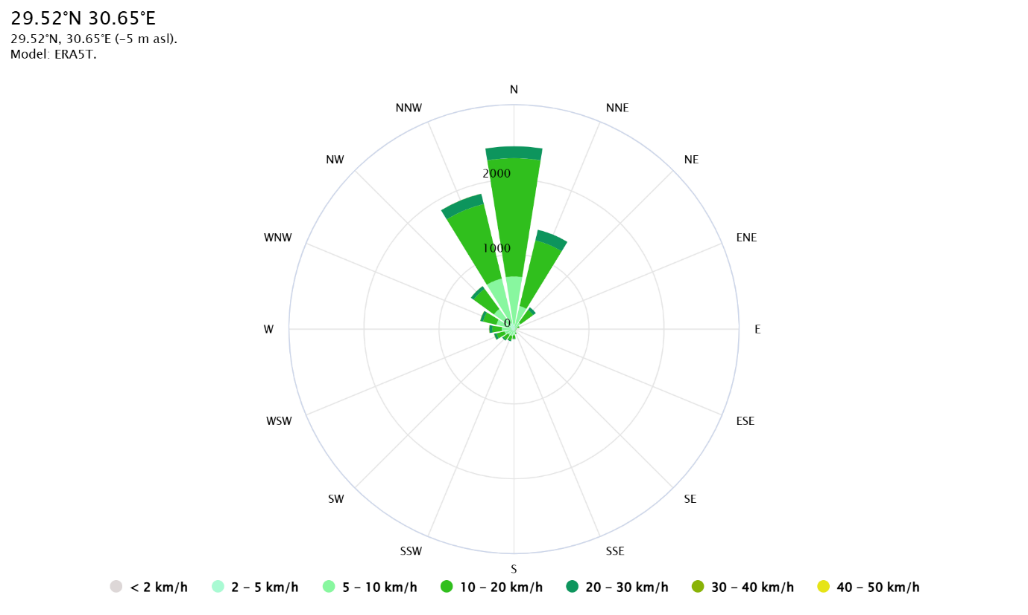


Fig. S8. Wind rose showing the wind direction of Fayoum.

**Water Resources and Agriculture**

In the northwestern part of the North Coast, fig orchards are widespread, typically relying on seasonal rainfall [S7]. However, the agricultural lands along the North Coast near the Nile Delta and the northern coast of the Nile Delta depend primarily on Nile water, which reaches these lands through various irrigation canals [S8]. These lands are used to cultivate a variety of crops, including guava (*Psidium guajava*), wheat (*Triticum aestivum*), dates (*Phoenix dactylifera*), olives (*Olea europaea*), alfalfa (*Medicago sativa*), and mangoes (*Mangifera sp*).

The agricultural lands located on the Cairo-Alexandria Desert Road rely heavily on groundwater wells and treated water, with efforts underway to diversify irrigation sources [S9]. The following are among the most common crops grown in this region: Orange (*Citrus sinensis*), barley (*Hordeum vulgare*), olive (*Olea europaea*), wheat (*Triticum* *aestivum*), broad bean (*Vicia faba*), and grape (*Vitis vinifera*).

Siwa is entirely dependent on groundwater derived from the Nubian Sandstone Aquifer System, which consists of a sandstone deposit spanning early Paleozoic to Cretaceous age with depths ranging from 2500 – 3000 m [S10]. Recently, the number of wells dug by the Siwa people has increased. The agricultural system in Siwa is organized into orchard plots. These orchards are subdivided into irrigation basins. At the center of each basin, farmers cultivate perennial trees, primarily olive (*Olea europaea* L.) or date palm (*Phoenix dactylifera* L.). The areas between these trees are used for intercropping with annual species, including alfalfa (*Medicago sativa*), wheat (*Triticum aestivum*), onion (*Allium cepa*), tomato (*Solanum lycopersicum*), cucumber (*Cucumis sativus*), watermelon (*Citrullus lanatus*), pepper (*Capsicum frutescens*), eggplant (*Solanum melongena*), marrow (*Cucurbita pepo*), and jute mallow (*Corchorus olitorius*) [S11].

The primary source of irrigation water for the agricultural lands surrounding Lake Qarun is mainly agrarian drainage water, which is drained from the agricultural lands in Fayoum, supplemented by water from the Al-Bats and Al-Wadi drains [S12]. Among the most famous agrarian crops found in this region are wheat (*Triticum aestivum*), olives (*Olea europaea*), and alfalfa (*Medicago sativa*).

**Salinity Conditions**

Soil erosion, salinity, and sodicity hazards are serious problems on the northwest coast of Egypt, leading to reduced soil quality and increased degradation. Sand beaches and sabkha units are characterized by high environmental hazards of both water erosion, salinity, and sodicity [S13]. Regarding climate change and its impacts on soil characteristics along the north coast of the Nile Delta, a rapid increase in salinity values during the past three decades was found. This increase may be due to the intrusion of salty water of the Mediterranean. On the other hand, organic matter content decreased due to higher temperatures, especially during the summer season [S14].

Water scarcity in arid and semiarid regions has resulted in the use of low-quality water for crop irrigation, as seen in agricultural lands near the Cairo-Alexandria Desert Road. The results demonstrated a significant spatial relationship between waterlogging and EC, SAR, and Na% in irrigation groundwater. Long-term irrigation with high salinity groundwater led to increased soil salinity, low soil permeability, and waterlogging. This study offers a time- and cost-efficient geospatial method for regional monitoring of surface waterlogging and mitigation strategies for cropland degradation and agricultural drainage water recycling that would benefit stakeholders and decision-makers [S15].

Since the year 2000, the Siwa region has experienced a marked increase in the rate of soil salinization and a corresponding decline in vegetation viability. This accelerated environmental change is attributable to a combination of factors, including a trend toward relative climate warming, the post-2000 installation of functionally inadequate drainage systems, and the lack of an integrated and effective water resource management framework [S16]. Between 1992 and 2015, the agricultural land area in the Siwa Oasis expanded from 22 km² to 81 km². This significant increase in cultivated area was accompanied by a concomitant rise in the extent of saline soils, from 35 km² to 64 km², and waterlogged areas, from 19 km² to 51 km². The observed land degradation is primarily driven by the expansion of reclamation projects, increased crop irrigation, and insufficient drainage infrastructure. Groundwater quality in the region shows degradation, with salinity measurements ranging from 2.28 to 5.45 dS m⁻¹ and an average of 3.76 dS m⁻¹. To ensure sustainable agriculture, the salt-affected soils of the oasis require a systematic reclamation program supported by the development of engineered irrigation and drainage systems [S17].

Lake Qarun constitutes a major physiographic element within the northern sector of the Fayoum depression. This enclosed lake occupies the lowest topographic point of the depression, covering approximately 53,000 acres with depths varying from 0.5 to 10.0 meters. Presently, the lake is brackish and exhibits high chlorosity, conditions which have led to the disappearance of most historically prevalent freshwater fish species. A daily inflow of roughly 100,000 cubic meters of water, carrying fine particulate matter, enters the lake. This volume represents excess irrigation water (amounting to 18% of the total water supplied to Fayoum Governorate), which discharges into the lake as agricultural drainage primarily via two major drains: El Batts on the eastern side and El Wadi on the western side. Several minor drains connected directly to the shoreline also contribute to this inflow. In recent years, Lake Qarun has undergone a pronounced change in water level, primarily attributed to variations in the rates of both drainage water inflow and evaporation. The average water level fluctuates between –43 and –45 m [S18]. Consequently, any further increase in drainage water volume would risk damaging adjacent agricultural soils [S19]. Therefore, Lake Qarun and its related adjacent soil sediments attract considerable scholarly attention due to their historical and scientific importance for studying this unique ecosystem. Awadallah [S20] reported that the degree of damage to neighbouring soil areas immediately adjacent to such an inland brackish lake is a controlling factor.

**Results and Discussion**

**Floristic** **Composition**

Table S2. Biodiversity indices (species richness, Species evenness, Shannon`s diversity index, and Simpson`s diversity index) of the studied stands

| Stand | species richness | Species evenness | Shannon`s diversity index | Simpson`s diversity index |
| --- | --- | --- | --- | --- |
| S1 | 19 | 0.606 | 1.784 | 0.6469 |
| S2 | 14 | 0.282 | 0.745 | 0.2618 |
| S3 | 16 | 0.804 | 2.228 | 0.8357 |
| S4 | 21 | 0.791 | 2.407 | 0.8682 |
| S5 | 11 | 0.675 | 1.619 | 0.7542 |
| S6 | 21 | 0.792 | 2.411 | 0.8691 |
| S7 | 11 | 0.794 | 1.903 | 0.8029 |
| S8 | 17 | 0.76 | 2.154 | 0.8395 |
| S9 | 7 | 0.709 | 1.38 | 0.6873 |
| S10 | 10 | 0.727 | 1.674 | 0.7617 |
| S11 | 18 | 0.194 | 0.561 | 0.2057 |
| S12 | 18 | 0.809 | 2.339 | 0.8524 |
| S13 | 14 | 0.824 | 2.174 | 0.8517 |
| S14 | 21 | 0.784 | 2.388 | 0.8555 |
| S15 | 10 | 0.413 | 0.95 | 0.3909 |
| S16 | 15 | 0.637 | 1.725 | 0.7378 |
| S17 | 17 | 0.605 | 1.715 | 0.7181 |
| S18 | 12 | 0.778 | 1.933 | 0.8008 |
| S19 | 18 | 0.658 | 1.901 | 0.7775 |
| S20 | 14 | 0.732 | 1.932 | 0.8082 |
| S21 | 16 | 0.586 | 1.625 | 0.6977 |
| S22 | 11 | 0.719 | 1.725 | 0.7601 |
| S23 | 14 | 0.578 | 1.525 | 0.6797 |
| S24 | 14 | 0.511 | 1.348 | 0.5505 |
| S25 | 11 | 0.557 | 1.336 | 0.6587 |
| S26 | 10 | 0.84 | 1.934 | 0.8241 |
| S27 | 11 | 0.787 | 1.887 | 0.7999 |
| S28 | 9 | 0.741 | 1.628 | 0.7528 |
| S29 | 14 | 0.705 | 1.86 | 0.7766 |
| S30 | 17 | 0.712 | 2.017 | 0.8247 |
| S31 | 11 | 0.858 | 2.057 | 0.8324 |
| S32 | 17 | 0.481 | 1.363 | 0.4944 |
| S33 | 15 | 0.79 | 2.139 | 0.854 |
| S34 | 14 | 0.821 | 2.166 | 0.8625 |
| S35 | 12 | 0.801 | 1.989 | 0.8285 |
| S36 | 13 | 0.82 | 2.104 | 0.849 |
| S37 | 12 | 0.279 | 0.693 | 0.2588 |
| S38 | 10 | 0.239 | 0.551 | 0.2028 |
| S39 | 11 | 0.211 | 0.505 | 0.181 |
| S40 | 12 | 0.26 | 0.646 | 0.2287 |
| S41 | 12 | 0.29 | 0.72 | 0.2773 |
| S42 | 7 | 0.19 | 0.37 | 0.1361 |
| S43 | 8 | 0.3 | 0.625 | 0.2538 |
| S44 | 5 | 0.753 | 1.212 | 0.6773 |
| S45 | 13 | 0.796 | 2.042 | 0.8143 |
| S46 | 10 | 0.741 | 1.706 | 0.7579 |
| S47 | 7 | 0.78 | 1.518 | 0.7266 |
| S48 | 11 | 0.737 | 1.767 | 0.7763 |
| S49 | 13 | 0.441 | 1.13 | 0.4374 |
| S50 | 6 | 0.749 | 1.343 | 0.6982 |
| S51 | 8 | 0.805 | 1.674 | 0.7765 |
| S52 | 9 | 0.473 | 1.039 | 0.4375 |
| S53 | 9 | 0.718 | 1.577 | 0.7194 |
| S54 | 17 | 0.849 | 2.406 | 0.8751 |
| S55 | 10 | 0.826 | 1.902 | 0.8095 |
| S56 | 7 | 0.795 | 1.546 | 0.7481 |
| S57 | 10 | 0.799 | 1.84 | 0.7992 |
| S58 | 16 | 0.867 | 2.405 | 0.8751 |
| S59 | 15 | 0.859 | 2.326 | 0.8672 |
| S60 | 12 | 0.847 | 2.106 | 0.8405 |
| S61 | 11 | 0.82 | 1.965 | 0.8169 |
| S62 | 15 | 0.878 | 2.377 | 0.8711 |
| S63 | 12 | 0.859 | 2.134 | 0.847 |
| S64 | 15 | 0.816 | 2.21 | 0.8428 |
| S65 | 8 | 0.772 | 1.604 | 0.7543 |
| S66 | 10 | 0.825 | 1.901 | 0.8086 |
| S67 | 14 | 0.268 | 0.707 | 0.2417 |
| S68 | 9 | 0.22 | 0.484 | 0.1796 |
| S69 | 11 | 0.341 | 0.819 | 0.316 |
| S70 | 7 | 0.816 | 1.589 | 0.7594 |
| Average | 12.5 | 0.656 | 1.629 | 0.6712 |

Table S3. Scientific and Common English Name of the recorded species

| Scientific Name | Common English Name |
| --- | --- |
| *Achillea fragrantissima* | Lavender Cotton |
| *Alhagi graecorum* | Camel thorn |
| *Amaranthus caudatus* | Love-lies-bleeding |
| *Anagallis arvensis* | Scarlet pimpernel |
| *Arthrocnemum macrostachyum* | Glaucous glasswort |
| *Astragalus incanus* | Hoary Milkvetch |
| *Astragalus spinosus* | Spiny milk-vetch |
| *Atriplex halimus* | Sea orache / Shrubby orache |
| *Avena fatua* | Wild oat |
| *Bassia indica* | Indian bassia |
| *Beta vulgaris* | Beet / Sugar beet |
| *Brassica tournefortii* | Asian mustard / Tournefort's mustard |
| *Bromus diandrus* | Great brome / Ripgut brome |
| *Calendula officinalis* | Pot marigold |
| *Calotropis procera* | Apple of Sodom / Giant milkweed |
| *Capsella bursa-pastoris* | Shepherd's purse |
| *Carduus getulus* | Moroccan thistle |
| *Centaurea aegyptiaca* | Egyptian starthistle |
| *Centaurea calcitrapa* | Red starthistle / Purple starthistle |
| *Chenopodium murale* | Nettle-leaved goosefoot |
| *Cichorium endivia* | Endive |
| *Convolvulus althaeoides* | Mallow bindweed |
| *Convolvulus hystrix* | - |
| *Convolvulus arvensis* | Field bindweed |
| *Conyza bonariensis* | Flax-leaved fleabane |
| *Cornulaca monacantha* | - |
| *Coronopus squamatus* | Swine wartcress |
| *Cressa cretica* | - |
| *Cynanchum acutum* | - |
| *Cynodon dactylon* | Bermuda grass |
| *Cyperus rotundus* | Purple nutsedge |
| *Desmostachya bipinnata* | Halfa grass / Big cordgrass |
| *Deverra tortuosa* | - |
| *Diplotaxis muralis* | Annual wall-rocket |
| *Echinochloa crus-galli* | Cockspur grass / Barnyard grass |
| *Echinops spinosus* | Globe thistle |
| *Emex spinosa* | Spiny emex / Devil's thorn |
| *Erodium laciniatum* | Cut-leaved stork's bill |
| *Eruca sativa* | Rocket / Arugula |
| *Euphorbia helioscopia* | Sun spurge |
| *Euphorbia peplus* | Petty spurge |
| *Fagonia arabica* | - |
| *Foeniculum vulgare* | Fennel |
| *Glebionis coronaria* | Crown daisy |
| *Glycyrrhiza glabra* | Licorice |
| *Haloxylon salicornicum* | - |
| *Heliotropium ovalifolium* | - |
| *Hordeum marinum* | Sea barley |
| *Imperata cylindrica* | Cogongrass |
| *Ipomoea cairica* | Cairo morning glory |
| *Juncus rigidus* | Sea rush |
| *Launaea nudicaulis* | - |
| *Lolium perenne* | Perennial ryegrass |
| *Lotus glaber* | Narrow-leaf bird's-foot trefoil |
| *Lycium europaeum* | European boxthorn |
| *Malva parviflora* | Cheeseweed / Small-flowered mallow |
| *Matthiola livida* | - |
| *Medicago sativa* | Alfalfa / Lucerne |
| *Melilotus indicus* | Annual yellow sweetclover |
| *Mentha longifolia* | Wild mint |
| *Mesembryanthemum crystallinum* | Common ice plant |
| *Mesembryanthemum nodiflorum* | Slender-leaf ice plant |
| *Nicotiana glauca* | Tree tobacco |
| *Onopordum alexandrinum* | Egyptian cotton thistle |
| *Oxalis corniculata* | Creeping woodsorrel |
| *Pancratium maritimum* | Sea daffodil |
| *Phoenix dactylifera* | Date palm |
| *Phragmites australis* | Common reed |
| *Plantago major* | Greater plantain |
| *Plantago squarrosa* | - |
| *Pluchea dioscoridis* | - |
| *Polypogon monspeliensis* | Annual beard grass |
| *Pulicaria undulata* | - |
| *Reichardia tingitana* | False sowthistle |
| *Reseda pruinosa* | - |
| *Rumex dentatus* | Toothed dock |
| *Rumex vesicarius* | Bladder dock |
| *Senecio vulgaris* | Common groundsel |
| *Senecio glaucus* | - |
| *Setaria viridis* | Green foxtail |
| *Silybum marianum* | Milk thistle |
| *Sisymbrium irio* | London rocket |
| *Solanum nigrum* | Black nightshade |
| *Solanum villosum* | Hairy nightshade |
| *Sonchus maritimus* | - |
| *Sonchus oleraceus* | Common sowthistle |
| *Sorghum halepense* | Johnson grass |
| *Spergularia marina* | Sand spurrey |
| *Suaeda pruinosa* | - |
| *Tamarix nilotica* | Nile tamarisk |
| *Thymelaea hirsuta* | - |
| *Tribulus terrestris* | Puncturevine |
| *Trigonella arabica* | - |
| *Typha domingensis* | Southern cattail |
| *Urospermum picroides* | - |
| *Urtica urens* | Annual nettle / Dwarf nettle |
| *Vaccaria pyramidata* | Cowherb |
| *Withania somnifera* | Winter cherry / Ashwagandha |
| *Xanthium spinosum* | Spiny cocklebur |
| *Zilla spinosa* | - |
| *Zygophyllum album* | - |
| *Zygophyllum coccineum* | - |

**References**

S1. Embabi, N.S. (2018). *Landscapes and landforms of Egypt*. Springer. 335p.

S2. Zahran, M.A. and Willis, A.J. (2008). *The vegetation of Egypt* (Vol. 2). Springer Science & Business Media. 451 p.

S3. Misak, R. F., Baki, A. A. and El–Hakim, M. S. (1997). On the causes and control of the waterlogging phenomenon, Siwa Oasis, northern Western Desert, Egypt. J. Arid Env., 37 (1), 23–32.

S4. Abd El–Ghani, M. M. and Fawzy, M. A. (2006). Plant diversity around springs and wells in five oases of the western desert, Egypt. Int. J. of Agr. & Bio., 8, 249–255.

S5. Shahin, M. (2007). *Water resources and hydrometeorology of the arab region* (Vol. 59). Springer Science and Business Media, 517 p.

S6. Vivian, C. (2004). *The western desert of Egypt*: An Explorer’s handbook. The American University in Cairo Press, 426 p.

S7. Abdel Ghaffar, M. K., Shoman, M. M., El Ghonamey,Y. K., and Abdellatif, A. D. (2019). Watershed modeling for water resource management in two basins of Northwestern Coastal Zone, Egypt, International Journal of Advanced Research, 7 (8), 33-47.

S8. Mohamed, N.N. (2016). Land degradation in the Nile Delta. In The Nile Delta (pp. 235-264). Cham: Springer International Publishing.

S9. Mohamed, N.N., 2021. Optimum economic uses of precious costly ground water in marginal and desert lands; Case study in Egypt. In Groundwater in Egypt’s Deserts (pp. 373-393). Cham: Springer International Publishing.

10 Aql, M. E. (1992). Nile valley between Sohag and Assiut, geomorphological study. Ph.D. Thesis, Faculty of Arts, Alexandria University, 338 p. [In Arabic].

S11. Bedair, R. (2016). Assessment of Vegetation and Environmental Factors Affecting Siwa Oasis Habitats, Western Desert, Egypt. Master's Thesis, Botany and Microbiology Department, Faculty of Science, Al-Azhar University, 202p.

S12. Khalil, M.T., Fishar, M.R., Shakir, S.H., Amer, A. and Nassif, M.G. (2017). Impact of drainage water on macrobenthos structure of Lake Qaroun, El-Fayoum, Egypt. Egyptian Journal of Aquatic Biology and Fisheries, 21(2), pp.17-32.

S13. Mohamed, E.S., Schütt, B. and Belal, A. (2013). Assessment of environmental hazards in the north western coast-Egypt using RS and GIS. *The Egyptian Journal of Remote Sensing and Space Science*, *16*(2), pp.219-229.

S14. El-Nahry, A.H. and Doluschitz, R. (2010). Climate change and its impacts on the coastal zone of the Nile Delta, Egypt. *Environmental Earth Sciences*, *59*(7), pp.1497-1506.

S15. Amer, R. (2021). Spatial relationship between irrigation water salinity, waterlogging, and cropland degradation in the arid and semi-arid environments. *Remote Sensing*, *13*(6), p.1047.

S16. Masoud, A.A. and Koike, K. (2006). Arid land salinization detected by remotely-sensed landcover changes: A case study in the Siwa region, NW Egypt. *Journal of arid environments*, *66*(1), pp.151-167.

S17. Elnaggar, A., El-Hamidi, K., Mousa, M. and Albakry, M. (2017). Mapping soil salinity and evaluation of water quality in Siwa Oasis using GIS. *Journal of Soil Sciences and Agricultural Engineering*, *8*(1), pp.9-19.

S18. Meshal, A.H. (1973). Water and salt budget of Lake Qarun, Fayoum, Egypt. Ph. D. Thesis, Alexandria Univ., Egypt. 109pp.

S19. Bishai, H.M. and S.Y. Kirollus (1980). The water budget of Lake Qarun and its physio-chemical characteristics. Water Supply and Management, Pergamon Press Ltd, Printed in Great Britain, 4: 93-97.

S20. Awadallah, A.A. (2004). Characterization of some wetland areas developed under arid and semi-arid climatic conditions of Egypt. Egypt. J of Appl. Sci., 19 (11): 437-460.
